# Supplementary material for: Influence of vitamin D supplementation on growth, body composition, pubertal development and spirometry in South African schoolchildren: a randomised controlled trial (ViDiKids)
Source: BMJ Paediatr Open. 2024 Apr 10;8(1):e002495. doi: 10.1136/bmjpo-2024-002495 (PMC11015302; doi:10.1136/bmjpo-2024-002495)
Supplement: Author's manuscript [file bmjpo-2024-002495.draft_revisions.pdf]

BMJ Paediatrics Open

BMJ Paediatrics Open is committed to open peer review. As part of this commitment we make the peer review history of every article we publish publicly available.

When an article is published we post the peer reviewers’ comments and the authors’ responses online. We also post the versions of the paper that were used during peer review. These are the versions that the peer review comments apply to.

The versions of the paper that follow are the versions that were submitted during the peer review process. They are not the versions of record or the final published versions. They should not be cited or distributed as the published version of this manuscript.

BMJ Paediatrics Open is an open access journal and the full, final, typeset and author-corrected version of record of the manuscript is available on our site with no access controls, subscription charges or pay-per-view fees (http://bmjpaedsopen.bmj.com).

If you have any questions on BMJ Paediatrics Open’s open peer review process please email info.bmjpo@bmj.com

BMJ Paediatrics Open

Influence of vitamin D supplementation on growth, body composition, pubertal development and spirometry in South African schoolchildren: a randomised controlled trial (ViDiKids)

Journal:	*BMJ Paediatrics Open*
Manuscript ID	bmjpo-2024-002495
Article Type:	Original research
Date Submitted by the Author:	07-Jan-2024
Complete List of Authors:	Middelkoop, Keren; University of Cape Town; University of Cape Town, Department of Medicine<br>Micklesfield, Lisa; Faculty of Health Sciences University of Cape Town, Department of Human Biology; Faculty of Health Sciences University of the Witwatersrand, Department of Paediatrics<br>Stewart, Justine; University of Cape Town; University of Cape Town, Department of Medicine<br>Walker, Neil; Queen Mary University of London<br>Jolliffe, David A; Queen Mary University of London<br>Mendham, Amy E; Faculty of Health Sciences University of Cape Town, Department of Human Biology; Faculty of Health Sciences University of the Witwatersrand, Department of Paediatrics<br>Coussens, Anna K; Faculty of Health Sciences University of Cape Town; Walter and Eliza Hall Institute of Medical Research<br>Nuttall, James; University of Cape Town, the Department of Paediatrics and Child Health<br>Momand, Waheedullah; Queen Mary University of London<br>Wilkinson, Robert J; Faculty of Health Sciences University of Cape Town; Francis Crick Institute<br>Bekker, Linda-Gail; University of Cape Town; University of Cape Town, Department of Medicine<br>Tang, Jonathan ; University of East Anglia Faculty of Medicine and Health Sciences, Department of Medicine Norwich Medical School, Faculty of Medicine and Health Sciences, University of East Anglia, Norwich Research Park,<br>Fraser, William; Norwich Medical School, University of East Anglia, Faculty of Medicine and Health Sciences<br>cooper, cyrus; University of Southampton<br>Harvey, Nick; Mr.C Epidemiology Resource Centre<br>Martineau, Adrian; Queen Mary University of London
Keywords:	Adolescent Health, Growth

I, the Submitting Author has the right to grant and does grant on behalf of all authors of the Work (as defined in the below author licence), an exclusive licence and/or a non-exclusive licence for contributions from authors who are: i) UK Crown employees; ii) where BMJ has agreed a CC-BY licence shall apply, and/or iii) in accordance with the terms applicable for US Federal Government officers or employees acting as part of their official duties; on a worldwide, perpetual, irrevocable, royalty-free basis to BMJ Publishing Group Ltd ("BMJ") its licensees and where the relevant Journal is co-owned by BMJ to the co-owners of the Journal, to publish the Work in this journal and any other BMJ products and to exploit all rights, as set out in our licence.

The Submitting Author accepts and understands that any supply made under these terms is made by BMJ to the Submitting Author unless you are acting as an employee on behalf of your employer or a postgraduate student of an affiliated institution which is paying any applicable article publishing charge ("APC") for Open Access articles. Where the Submitting Author wishes to make the Work available on an Open Access basis (and intends to pay the relevant APC), the terms of reuse of such Open Access shall be governed by a Creative Commons licence – details of these licences and which Creative Commons licence will apply to this Work are set out in our licence referred to above.

Other than as permitted in any relevant BMJ Author's Self Archiving Policies, I confirm this Work has not been accepted for publication elsewhere, is not being considered for publication elsewhere and does not duplicate material already published. I confirm all authors consent to publication of this Work and authorise the granting of this licence.

Confidential: For Review Only

Influence of vitamin D supplementation on growth, body composition, pubertal development and spirometry in South African schoolchildren: a randomised controlled trial (ViDiKids)

Keren Middelkoop[1,2*]
Lisa K Micklesfield[3,4*]
Justine Stewart[1,2]
Neil Walker[5]
David A Jolliffe[6]
Amy E Mendham[3,4]
Anna K Coussens[7,8]
James Nuttall[9]
Jonathan C Y Tang[10,11]
William D Fraser[10,11]
Waheedullah Momand[6]
Cyrus Cooper[12,13]
Nicholas C Harvey[12,13]
Robert J Wilkinson[7,14,15]
Linda-Gail Bekker[1,2]
Adrian R Martineau[6†]

1, Desmond Tutu HIV Centre, Institute of Infectious Disease & Molecular Medicine, University of Cape Town, Observatory, South Africa
2, Department of Medicine, University of Cape Town, Observatory, South Africa
3, Health through Physical Activity, Lifestyle and Sport Research Centre (HPALS), Division of Physiological Sciences, Department of Human Biology, Faculty of Health Sciences, University of Cape Town, Cape Town, South Africa
4, SAMRC/Wits Developmental Pathways for Health Research Unit, Department of Paediatrics, Faculty of Health Sciences, University of the Witwatersrand, Johannesburg, South Africa
5, Wolfson Institute of Population Health, Barts and The London School of Medicine and Dentistry, Queen Mary University of London, London, UK
6, Blizard Institute, Barts and The London School of Medicine and Dentistry, Queen Mary University of London, London, UK

Confidential: For Review Only

7, Centre for Infectious Diseases Research in Africa and Institute of Infectious Disease and Molecular Medicine, Faculty of Health Sciences, University of Cape Town, Observatory 7925, South Africa

8, Infectious Diseases and Immune Defence Division, Walter and Eliza Hall Institute of Medical Research, Parkville, Australia

9, Paediatric Infectious Diseases Unit, Red Cross War Memorial Children's Hospital and the Department of Paediatrics and Child Health, University of Cape Town, Cape Town, South Africa

10, Norwich Medical School, University of East Anglia, Norwich Research Park, Norwich, UK

11, Departments of Laboratory Medicine, Clinical Biochemistry and Departments of Diabetes and Endocrinology, Norfolk and Norwich University Hospital NHS Foundation Trust, Norwich, UK

12, MRC Lifecourse Epidemiology Centre, University of Southampton, Southampton UK

13, NIHR Southampton Biomedical Research Centre, University of Southampton, Southampton UK and University Hospital Southampton NHS Foundation Trust, Southampton, UK

14, The Francis Crick Institute, London, NW1 1AT, UK

15, Department of Infectious Diseases, Imperial College London, London, W12 0NN, UK

* These authors contributed equally

†To whom correspondence should be addressed at the Blizard Institute, Barts and The London School of Medicine and Dentistry, Queen Mary University of London, 4 Newark St, London E1 2AT, UK; Tel: +44 207 882 7242; Email: a.martineau@qmul.ac.uk

Confidential: For Review Only

ABSTRACT

Objective: To determine whether weekly oral vitamin D supplementation influences growth, body composition, pubertal development or spirometric outcomes in South African schoolchildren.

Design: Phase 3 double-blind randomised placebo-controlled trial (clinicaltrials.gov registration no. NCT02880982).

Setting: Socio-economically disadvantaged peri-urban district of Cape Town, South Africa

Participants: 1682 children of Black African ancestry attending government primary schools and aged 6-11 years at baseline.

Interventions: Oral vitamin D_3 (10,000 IU/week) vs. placebo for 3 years

Main outcome measures: height-for-age and body mass index-for-age, measured in all participants); Tanner scores for pubertal development, spirometric lung volumes and body composition, measured in a subset of 450 children who additionally took part in a nested sub-study.

Results: Mean serum 25-hydroxyvitamin D_3 concentration at 3-year follow-up was higher among children randomised to receive vitamin D vs. placebo (104.3 vs. 64.7 nmol/L, respectively; mean difference [MD] 39.7 nmol/L, 95% CI 37.6 to 41.9 nmol/L). No statistically significant differences in height-for-age z-score (adjusted MD [aMD] -0.08, 95% CI -0.19 to 0.03) or body mass index-for-age z-score (aMD -0.04, 95% CI -0.16 to 0.07) were seen between vitamin D vs. placebo groups at follow-up. Among sub-study participants, allocation to vitamin D vs. placebo did not influence pubertal development scores, % predicted forced expiratory volume in 1 second (FEV1), % predicted forced vital capacity (FVC), % predicted FEV1/FVC, fat mass or fat-free mass.

Conclusions: Weekly oral administration of 10,000 IU vitamin D_3 boosted vitamin D status but did not influence growth, body composition, pubertal development or spirometric outcomes in South African schoolchildren.

Confidential: For Review Only

KEY MESSAGES

What is already known on this topic?

- Observational studies have reported independent associations between vitamin D deficiency in childhood and slower linear growth, reduced lean mass, obesity and precocious puberty.
- A phase 2 clinical trial conducted in Mongolia reported that a 6-month course of vitamin D supplementation increased height gain in 113 vitamin D deficient schoolchildren aged 12-15 years; however, these results were not confirmed by a recent phase 3 trial conducted in the same setting.
- RCTs to determine effects of vitamin D supplementation on growth and development in schoolchildren have not been conducted in other settings.

What this study adds

- This placebo-controlled phase 3 clinical trial, conducted in 1682 Black African schoolchildren in Cape Town, South Africa, showed that a 3-year course of weekly vitamin D supplementation was effective in elevating circulating 25-hydroxyvitamin D concentrations.
- However, this was not associated with any effect on linear growth, body composition, pubertal development or spirometric lung volumes.

How this study might affect research, practice or policy

- Our findings do not support use of vitamin D supplementation as an intervention to influence child growth, body composition, pubertal development or spirometric lung volumes.

Confidential: For Review Only

INTRODUCTION

Middle childhood and early adolescence represent key periods for growth and development that has an important influence on stature and health outcomes in later adolescence and adulthood.[1] Stunting in childhood has long been recognised to associate with multiple adverse long-term health outcomes,[2] and is particularly common in lower-income countries.[3] These settings have also witnessed an emerging epidemic of childhood obesity,[4] which has in turn been associated with accelerated pubertal development.[5] Interventions to alleviate these public health challenges are urgently needed.

Vitamin D is a fat-soluble micronutrient with pleotropic effects on human health.[6] Observational studies have reported that vitamin D deficiency, as evidenced by low circulating concentrations of its major metabolite 25-hydroxyvitamin D (25[OH]D), associates with slower linear growth,[7] reduced lean mass,[8] childhood obesity[9-11] and precocious puberty,[12,13] potentially reflecting the ability of vitamin D to stimulate production of insulin-like growth factor 1[14] and regulate adipogenesis.[15] A phase 2 randomised controlled trial (RCT) conducted in Mongolia reported that a 6-month course of vitamin D supplementation increased height gain in 113 vitamin D deficient schoolchildren aged 12-15 years,[16] but these results were not confirmed by a recent phase 3 RCT conducted in the same setting.[17] RCTs to determine effects of vitamin D supplementation on growth and development in schoolchildren have not been conducted in other settings representing children at different risks of malnutrition, stunting and obesity. An opportunity to conduct such an investigation recently arose as part of the ViDiKids trial, a multicentre phase 3 RCT which investigated the effects of weekly oral administration of 10,000 IU vitamin D_3 for 3 years on the primary outcome of tuberculosis infection in a cohort of 1682 schoolchildren aged 6-11 years living in a socio-economically disadvantaged peri-urban district of Cape Town, South Africa.[18] Height-for-age z-scores and body mass index-for age z-scores were assessed in all participants (n=1682), and Tanner scores for pubertal development, spirometric lung volumes and body composition were assessed in a subset of 450 children who also took part in a nested sub-study.

Confidential: For Review Only

METHODS

TRIAL DESIGN, SETTING, APPROVALS AND REGISTRATION

We conducted a multicentre phase 3 double-blind individually randomised placebo-controlled trial of weekly oral vitamin D supplementation in 23 government schools in Cape Town, South Africa, as previously described.[18,19] The primary outcome was acquisition of tuberculosis infection, as evidenced by conversion of a QuantiFERON-TB Gold Plus (QFT-Plus) assay result from negative at baseline to positive at 3-year follow-up. The current manuscript reports effects of the intervention on pre-specified secondary outcomes relating to growth in all study participants, and body composition, pubertal development and spirometry in a subset of participants who additionally took part in a nested sub-study. The trial was sponsored by Queen Mary University of London and registered on the South African National Clinical Trials Register (DOH-27-0916-5527) and ClinicalTrials.gov (ref NCT02880982).

RESEARCH ETHICS APPROVAL

The trial was approved by the University of Cape Town Faculty of Health Sciences Human Research Ethics Committee (Ref: 796/2015) and the London School of Hygiene and Tropical Medicine Observational/Interventions Research Ethics Committee (Ref: 7450-2)

PARTICIPANTS

Inclusion criteria for the main trial were enrolment in Grades 1-4 at a participating school; age 6 to 11 years at screening; and written informed assent / consent to participate in the main trial provided by children and their parent / legal guardian, respectively. Exclusion criteria for the main trial were a history of previous tuberculosis infection, active TB disease or any chronic illness other than asthma (including known or suspected HIV infection) prior to enrolment; use of any regular medication other than asthma medication; use of vitamin D supplements at a dose of more than 400 IU/day in the month before enrolment; plans to move away from study area within 3 years of enrolment; inability to swallow a placebo soft gel capsule with ease; and clinical evidence of rickets or a positive QFT-Plus assay result at screening. An

Confidential: For Review Only

additional inclusion criterion for the sub-study was enrolment in Grade 4 at a participating school.

ENROLMENT

Full details of enrolment procedures are described in Supplementary Methods (Supplementary Material). Eligible participants underwent measurement of weight using a digital floor scale (Charder Medical, Taichung City, Taiwan), height using a portable stadiometer (HM200P, Charder Medical) and waist circumference using a measuring tape. Sub-study participants also underwent spirometry according to ERS/ATS standards[20] using a portable spirometer (Carefusion, San Diego, CA) and measurement of body composition by dual energy x-ray absorptiometry (DXA) as described below.

RANDOMISATION AND BLINDING

Full details of randomisation and blinding procedures have been described previously,[18,19] and are presented in Supplementary Methods (Supplementary Material). Briefly, eligible and assenting children whose parents consented to their participation in the trial were individually randomised to receive a weekly capsule containing vitamin D_3 or placebo for three years, with a one-to-one allocation ratio and randomisation stratified by school of attendance. Treatment allocation was concealed from participants, care providers and all trial staff (including senior investigators and those assessing outcomes) until completion of the trial to maintain the double-blind.

INTERVENTION

Study medication comprised a 3-year course of weekly soft gel capsules manufactured by the Tishcon Corporation (Westbury, NY, USA), containing either 0.25 mg (10,000 international units) cholecalciferol (vitamin D_3) in olive oil (intervention arm) or olive oil without any vitamin D_3 content (placebo arm). Active and placebo capsules had identical appearance and taste. Capsules were taken under direct observation of study staff during school term time. Further details of administration of study medication are provided in Supplementary Methods (Supplementary Material).

Confidential: For Review Only

FOLLOW-UP ASSESSMENTS

At 1-year, 2-year and 3-year follow-up, height, weight and waist circumference were measured as at baseline. At 3-year follow-up, sub-study participants were additionally invited to undergo repeat spirometry and DXA scanning, and to complete a Tanner self-assessment questionnaire for pubertal development.[21]

OUTCOMES

The primary outcome for the main trial, reported elsewhere,[18] was the QFT-Plus result at the end of the study. The following secondary outcomes were assessed for all participants: height-for-age, BMI-for-age, waist circumference-for-age and waist-to-height ratio (all participants). Additional outcomes assessed in sub-study participants only were: whole body fat mass, fat-free soft tissue mass, % predicted forced vital capacity (FVC), % predicted forced expiratory volume in one second (FEV1), % predicted FEV1/FVC, mean Tanner scores for pubic hair (males and females), external genitalia (males only) or breast development (females only), the proportion of participants reaching menarche by the end of the trial (females only) and mean age at menarche (females who reached menarche by the end of the trial only).

DXA

Body composition (whole body fat mass and fat-free soft tissue mass) was assessed using a Hologic Discovery-W® dual energy x-ray absorptiometry (DXA) scanner at the Sports Science Institute of South Africa, University of Cape Town. All scans were performed by a trained radiographer on one scanner (Hologic, Bedford, MA, USA) using standard procedures, and analysed using Apex software (Version 13.4.1). Quality assurance checks were carried out prior to scanning and generated coefficients of variation <0.5%.

LABORATORY ASSESSMENTS

Serum concentrations of 25(OH)D_3 were measured using liquid chromatography tandem mass spectrometry (LC-MS/MS) as previously described.[22] Further details are presented in Supplementary Methods (Supplementary Material).

Confidential: For Review Only

SAMPLE SIZE

Sample size for the main trial was predicated on power to detect an effect of the intervention on the primary outcome (the proportion of children with a positive QFT-Plus assay result at 3-year follow-up), as previously described.[18] Sample size for the sub-study was predicated on power to detect an effect of the intervention on bone mineral content, as previously described.[19]

STATISTICAL ANALYSES

Full details of statistical analyses are provided in Supplementary Methods (Supplementary Material). Briefly, effects of treatment on age- and sex-adjusted z-scores for anthropometric outcomes were estimated by fitting allocation to vitamin D vs. placebo as the sole fixed effect in a mixed effects linear regression model with a random effect for repeated assessments of each individual participant and a random effect of school of attendance. Effects of treatment on DXA and spirometric outcomes were analysed using multi-level mixed models with adjustment for baseline values and a random effect for school. Tanner metrics were analysed in a similar fashion, but restricted to the applicable sex and without baseline adjustment. Pre-specified sub-group analyses were conducted to determine whether the effect of vitamin D supplementation was modified by sex (male vs. female), baseline deseasonalised 25(OH)D_3 concentration (<75 vs. ≥75 nmol/L)[24] and calcium intake (< vs. ≥ median value of 466 mg/day).[19] An Independent Data Monitoring Committee reviewed accumulating serious adverse event data at 6-monthly intervals, and recommended continuation of the trial at each review. No interim efficacy analysis was performed.

PATIENT AND PUBLIC INVOLVEMENT

The Desmond Tutu HIV Centre's Community Advisory Group were consulted on the design and conduct of the ViDiKids trial.

Confidential: For Review Only

RESULTS

PARTICIPANTS

A total of 2852 children were screened for eligibility from March 2017 to March 2019, of whom 2271 underwent QFT testing: 1682 (74.1%) QFT-negative children were randomly assigned to receive vitamin D_3 (829 participants) or placebo (853 participants) as previously described.[18] 450/1682 (26.8%) participants in the main trial also participated in the sub-study, of whom 228 vs. 222 participants were allocated to the vitamin D vs. placebo arms, respectively (Fig. 1). Table 1 presents baseline characteristics of children in the main trial and in the sub-study, overall and by study arm. Mean age was higher among participants in the sub-study vs. all those in the main trial (10.1 vs. 8.9 years, respectively), reflecting the fact that participation in the sub-study was restricted to children enrolled in Grade 4. Prevalence of obesity and stunting were lower in the sub-study vs. the main trial (3.8% vs 5.5%, and 6.4% vs. 11.1%, respectively. Baseline characteristics were otherwise well balanced for all participants in the main trial vs. those who additionally participated in the sub-study: 52.4% vs. 52.0% were female and mean serum 25(OH)D_3 concentrations were 71.2 nmol/L vs. 70.0 nmol/L. Within the main trial and the sub-study, baseline characteristics of those randomised to vitamin D vs. placebo were also well balanced. The median duration of follow-up was 3.16 years (interquartile range, 2.83 to 3.38 years) and was not different between the two study arms. For the main trial, mean serum 25(OH)D_3 concentrations at 3-year follow-up were higher among children randomised to receive vitamin D vs. placebo (104.3 vs. 64.7 nmol/L, respectively; mean difference 39.7 nmol/L, 95% CI for difference 37.6 to 41.9 nmol/L).

GROWTH OUTCOMES

Among participants in the main trial, allocation to vitamin D vs. placebo did not influence mean height-for-age z-scores at annual follow-up, either overall or within sub-groups defined by sex, calcium intake or baseline 25(OH)D concentration (Table 2: P values for interaction >0.05). Similarly, no effect of the intervention was seen on mean BMI-for-age z-scores (Table 3), waist circumference-for-age z-scores (Table S1) or waist-to-height ratio z-scores (Table S2), either overall or by sub-group (P

Confidential: For Review Only

values for interaction >0.05). Among sub-study participants, allocation to vitamin D vs. placebo did not influence fat mass or fat-free soft tissue mass (Table S3) or % predicted FEV1 or FEV1/FVC (Table S4), either overall or by sub-group. For the outcome of % predicted FVC, allocation to vitamin D vs. placebo did not have an effect overall, or within sub-groups defined by calcium intake or baseline 25(OH)D concentration; however, the P value for interaction associated with the sub-group analysis by sex (P=0.049) raised the possibility that this factor might modify the effect of vitamin D on % predicted FVC (Table S4).

DEVELOPMENTAL OUTCOMES

Among sub-study participants, no inter-arm differences were seen in mean Tanner scores for pubic hair (males and females), external genitalia (males only), proportion menstruating (females only), mean age at menarche (females only), or breast development (females only), either overall or within sub-groups defined by calcium intake or baseline 25(OH)D concentration (Table 4).

ADVERSE EVENTS

Incidence of adverse events by trial arm has been reported elsewhere.[18] No serious events arising in the trial were adjudged related to administration of vitamin D or placebo.

Confidential: For Review Only

DISCUSSION

We report findings from the first randomised controlled trial to investigate effects of vitamin D supplementation on growth and developmental indices in schoolchildren of Black African ancestry. Administration of oral vitamin D supplementation at a dose of 10,000 IU per week for 3 years was effective in boosting vitamin D status, but it did not impact linear growth, body mass index, body composition, spirometric lung volumes or self-assessed pubertal development, either overall or in sub-groups defined by sex, calcium intake or baseline vitamin D status.

Null findings from the current study contrast with those from observational studies reporting independent associations between vitamin D deficiency and reduced lean mass,[8] slower linear growth,[7] childhood obesity[9-11] and precocious puberty.[12] However, they are consistent with those of the only other phase 3 RCT to investigate effects of vitamin D on growth and development in school-age children that we are aware of, which reported no effect of weekly vitamin D supplementation on growth or development among Mongolian schoolchildren aged 6-13 years at baseline.[17] Contrasting findings from observational studies vs. clinical trials may reflect the fact that the former are more susceptible to effects of confounding or reverse causality. Although we observed limited evidence (P for interaction 0.049) to support the hypothesis that effects of vitamin D on FVC might be modified by sex, this finding may have arisen because of type 1 error, given the multiplicity of analyses conducted.

Our study has several strengths. The intervention was sustained (3 years), allowing ample time for any effects of vitamin D supplementation on outcomes of interest to manifest. Moreover, the intervention was effective in elevating serum 25(OH)D concentrations, reflecting adequacy of the dosing regimen employed as well as good adherence resulting from directly observed administration of weekly supplements in schools during termtime. Our large sample size and low rates of loss to follow-up maximised power to detect modest effects of the intervention, particularly for outcomes that were assessed in the main trial population. Furthermore, we assessed a broad range of anthropometric and developmental outcomes, that included use of DXA, the gold standard investigation for assessment of body composition.

Confidential: For Review Only

Our study also has some limitations. Baseline prevalence of vitamin D deficiency was low, perhaps reflecting plentiful exposure to sunshine in the study setting.[22] Our results cannot therefore be generalised to settings where vitamin D deficiency is common. However, we highlight that results from our trial in Mongolia (where baseline prevalence of vitamin D deficiency was much higher than we observed in the current study) were also null for outcomes of linear growth and body mass index.[17] Accordingly, null results from the current trial cannot be explained simply by the relatively high baseline vitamin D status of participants in Cape Town. A second potential limitation relates to the fact that pubertal status was assessed by participants themselves, rather than by health professionals, whose judgement may be more objective. However, the double-blind placebo-controlled trial design of our study will have distributed any resultant imprecision equally between study arms, ensuring that bias was not introduced. Moreover, pubertal self-assessment has previously been done by adolescent participants in another South African cohort study, and shown to be reliable.[25]

In conclusion, we report that oral vitamin D supplementation at a dose of 10,000 IU/week for 3 years was effective in elevating serum 25(OH)D concentrations in schoolchildren of Black African ancestry living in Cape Town, South Africa. However, this was not associated with any effects on linear growth, body habitus, spirometric outcomes or pubertal development.

ACKNOWLEDGEMENTS

This research was funded by the UK Medical Research Council (refs MR/R023050/1 and MR/M026639/1, both awarded to ARM). RJW was supported by Wellcome (104803, 203135). He also received support from the Francis Crick Institute which is funded by Cancer Research UK (FC2112), the UK Medical Research Council (FC2112) and Wellcome (FC2112). We thank all the children who participated in the trial, and their parents / guardians; members of the Independent Data Monitoring Committee (Prof Guy Thwaites, Oxford University Clinical Research Unit, Ho Chi Minh City, Vietnam [Chair]; Prof John Pettifor, University of the Witwatersrand, Johannesburg, South Africa; and Prof Sarah Walker, MRC Clinical Trials Unit, London,

Confidential: For Review Only

UK); and members of the Trial Steering Committee (Prof Beate Kampmann, London School of Hygiene and Tropical Medicine, London, UK [Chair]; Prof Ashraf Coovadia, University of the Witwatersrand, Johannesburg, South Africa; Dr Karen Jennings, City Health, Cape Town, South Africa; Dr Robin Dyers, Department of Health, Western Cape Government, Cape Town, South Africa; and Dr Guy de Bruyn, Sanofi Pasteur, Swiftwater PA USA). For the purposes of open access the author has applied a CC-BY public copyright to any author-accepted manuscript arising from this submission.

CONTRIBUTORS

ARM conceived the study. KM, LKM, AKC, JN, CC, NCH, RLH, RJW, LGB and ARM contributed to study design and protocol development. KM led on trial implementation, with support from JS, CD, DAJ, JN, LGB and ARM. LKM and AEM oversaw performance of DXA scans. JCYT and WDF performed and supervised the conduct of biochemical assays. NW, RLH and ARM drafted the statistical analysis plan. DAJ, KM, JS, NW, WM and CD managed data. NW accessed, verified and analysed the data underlying the study. ARM and DAJ wrote the first draft of the trial report. All authors made substantive comments thereon and approved the final version for submission.

COMPETING INTERESTS

ARM declares receipt of funding in the last 36 months to support vitamin D research from the following companies who manufacture or sell vitamin D supplements: Pharma Nord Ltd, DSM Nutritional Products Ltd, Thornton & Ross Ltd and Hyphens Pharma Ltd. ARM also declares receipt of vitamin D capsules for clinical trial use from Pharma Nord Ltd, Synergy Biologics Ltd and Cytoplan Ltd; support for attending meetings from Pharma Nord Ltd and Abiogen Pharma Ltd; receipt of consultancy fees from DSM Nutritional Products Ltd and Qiagen Ltd; receipt of a speaker fee from the Linus Pauling Institute; participation on Data and Safety Monitoring Boards for the VITALITY trial (Vitamin D for Adolescents with HIV to reduce musculoskeletal morbidity and immunopathology, Pan African Clinical Trials Registry ref PACTR20200989766029) and the Trial of Vitamin D and Zinc Supplementation for Improving Treatment Outcomes Among COVID-19 Patients in India (ClinicalTrials.gov ref NCT04641195);

Confidential: For Review Only

and unpaid work as a Programme Committee member for the Vitamin D Workshop. All other authors declare that they have no competing interests.

DATA AVAILABILITY STATEMENT

Anonymised data may be requested from the corresponding author to be shared subject to terms of research ethics committee approval.

Confidential: For Review Only

Table 1: Participants' baseline characteristics by allocation: main study and sub-study

		Main study (n=1682)			Sub-study (n=450 subset)		
		**Overall (n=1,682)**	**Vitamin D arm (n=829)**	**Placebo arm (n=853)**	**Overall (n=450)**	**Vitamin D arm (n=228)**	**Placebo arm (n=222)**
Mean age, years (s.d.)		8.9 (1.4)	8.9 (1.4)	8.8 (1.3)	10.1 (0.7)	10.2 (0.7)	10.0 (0.6)
Female sex, n (%)		880 (52.4)	437 (52.8)	443 (51.9)	234 (52.0)	116 (50.9)	118 (53.2)
Ethnic origin[(1)]	Xhosa, n (%)	1615 (97.9)	788 (97.3)	827 (98.5)	424 (96.4)	214 (96.0)	210 (96.8)
	Other, n (%)	35 (2.1)	22 (2.7)	13 (1.5)	16 (3.6)	9 (4.0)	7 (3.2)
Type of residence	Brick, n (%)	867 (51.5)	423 (51.0)	444 (52.1)	230 (51.1)	121 (53.1)	109 (49.1)
	Informal, n(%)	815 (48.5)	406 (49.0)	409 (47.9)	220 (48.9)	107 (46.9)	113 (50.9)
Parental education[(1,2)]	Primary school, n (%)	60 (3.6)	34 (4.1)	26 (3.1)	24 (5.3)	16 (7.0)	8 (3.6)
	Secondary school or higher, n (%)	1618 (96.4)	792 (95.9)	826 (96.9)	426 (94.7)	212 (93.0)	214 (96.4)
Mean monthly household income, 1,000 ZAR (s.d.)		1.88 (2.20)	1.81 (2.15)	1.95 (2.25)	1.57 (2.31)	1.52 (2.69)	1.63 (1.86)
Mean height-for-age z-score (s.d.)[(1)]		-0.55 (1.20)	-0.56 (1.26)	-0.54 (1.15)	-0.43 (0.96)	-0.43 (0.98)	-0.44 (0.95)
Height-for-age z-score category	<-2.00 (stunted), n (%)	186 (11.1)	99 (11.9)	87 (10.2)	29 (6.4)	16 (7.0)	13 (5.9)
	≥ -2.00, n (%)	1496 (88.9)	730 (88.1)	766 (89.8)	421 (93.6)	212 (93.0)	209 (94.1)
Mean BMI-for-age z-score (s.d.)[(1)]		0.33 (1.03)	0.31 (1.03)	0.34 (1.03)	0.20 (0.97)	0.19 (0.92)	0.21 (1.02)
BMI-for-age z-score category	<-2.00 (thin), n (%)	26 (1.5)	15 (1.8)	11 (1.3)	7 (1.6)	4 (1.8)	3 (1.4)
	> -2.00 & ≤1.00, n (%)	1223 (72.7)	602 (72.6)	621 (72.8)	354 (78.7)	178 (78.1)	176 (79.3)
	>1.00 & ≤2.00 (overweight), n (%)	340 (20.2)	165 (19.9)	175 (20.5)	72 (16.0)	42 (18.4)	30 (13.5)
	>2.00 (obese), n (%)	93 (5.5)	47 (5.7)	46 (5.4)	17 (3.8)	4 (1.8)	13 (5.9)
Mean waist circumference-for-age z-score (s.d.)[(1)]		-0.13 (0.90)	-0.11 (0.90)	-0.15 (0.91)	-0.20 (0.98)	-0.20 (0.98)	-0.20 (0.98)
Mean waist-to-height ratio z-score (s.d.)[(1)]		0.03 (0.85)	0.05 (0.83)	0.00 (0.86)	-0.05 (0.86)	-0.05 (0.85)	-0.04 (0.88)
Mean whole body fat mass, kg (s.d.)		--	--	--	3.71 (2.26)	3.66 (2.13)	3.77 (2.40)
Mean whole body fat-free soft tissue mass, kg (s.d.)		--	--	--	9.27 (1.95)	9.39 (2.02)	9.15 (1.86)
% predicted FEV1 (s.d.)		--	--	--	83.2 (15.7)	83.8 (15.2)	82.7 (16.2)
% predicted FVC (s.d.)		--	--	--	94.8 (18.7)	94.6 (18.2)	95.0 (19.3)
% predicted FEV1/FVC (s.d.)		--	--	--	91.4 (16.9)	92.2 (16.3)	90.5 (17.5)
Calcium intake[(1)]	<median, n (%)[(3)]	817 (50.0)	383 (47.6)	434 (52.3)	248 (56.2)	119 (53.6)	129 (58.9)
	≥median, n (%)[(3)]	817 (50.0)	421 (52.4)	396 (47.7)	193 (43.8)	103 (46.4)	90 (41.1)
Mean serum 25(OH)D_3 concentration, nmol/L (s.d.)[(1,4)]		71.2 (14.8)	71.2 (14.5)	71.1 (15.0)	70.0 (13.5)	70.4 (12.1)	69.6 (14.9)
Serum 25(OH)D_3 concentration, category[(1,4)]	<25 nmol/L, n (%)	1 (0.1)	0 (0.0)	1 (0.1)	1 (0.3)	0 (0)	1 (0.6)
	≥ 25 nmol/L & < 50 nmol/L, n (%)	74 (5.4)	34 (5.1)	40 (5.8)	20 (5.5)	7 (3.7)	13 (7.4)
	≥ 50 nmol/L & < 75 nmol/L, n (%)	787 (57.7)	394 (58.8)	393 (56.6)	214 (59.0)	114 (61.0)	100 (56.8)
	≥75 nmol/L, n (%)	502 (36.8)	242 (36.1)	260 (37.5)	128 (35.3)	66 (35.3)	62 (35.2)

Confidential: For Review Only

Abbreviations: $25(OH)D_3$, 25-hydroxyvitamin D_3. ALP, alkaline phosphatase. BMI, body mass index. BMC, bone mineral content. CTX, C-terminal cross-linked telopeptide; P1NP, serum procollagen type I N-propeptide. S.d., standard deviation. ZAR, South African Rand.

Footnotes. 1, missing data (for bone sub-study: ethnicity, n=5 vitamin D arm, n=5 placebo arm; for calcium intake, n=6 Vitamin D arm, n=3 placebo arm; for serum $25(OH)D_3$ concentration, n=66 vitamin D arm, n=62 placebo arm; for serum adjusted calcium and total ALP concentration, n=41 vitamin D arm, n=45 placebo arm; for PTH: n=42 vitamin D arm, n=46 placebo arm; for CTX: n=41 vitamin D arm, n=46 placebo arm; for P1NP: n=43 vitamin D arm, n=46 placebo arm; for fracture study: ethnicity, n=19 vitamin D arm, n=13 placebo arm; parental education, n=3 vitamin D arm, n=1 placebo arm; BMI-for-age z-score, n=2 vitamin D arm, n=0 placebo arm; height-for-age z-score, n=2 vitamin D arm, n=0 placebo arm; for calcium intake, n=25 Vitamin D arm, n=23 placebo arm; for serum $25(OH)D_3$ concentration, n=159 vitamin D arm, n=159 placebo arm; **2,** highest level of education of at least one parent. **3,** median calcium intake 466 mg/day. **4,** deseasonalised values

Confidential: For Review Only

Table 2. Mean height-for-age z-scores at annual follow-up by allocation, main study participants: overall and by sub-group

		Follow-up time point	Vitamin D arm: mean value (s.d.) [n]	Placebo arm: mean value (s.d.) [n]	Adjusted mean difference (95% CI)[1]	P for timepoint	Overall P	P for interaction
Overall		1 year	-0.22 (1.12) [664]	-0.15 (1.07) [661]	-0.06 (-0.17 to 0.06)	0.33		--
		2 years	-0.14 (1.05) [613]	-0.06 (1.01) [606]	-0.04 (-0.16 to 0.07)	0.46	0.22	
		3 years	-0.26 (1.04) [670]	-0.14 (1.05) [691]	-0.08 (-0.19 to 0.03)	0.17		
By sex	Boys	1 year	-0.31 (1.14) [304]	-0.27 (1.06) [306]	-0.02 (-0.17 to 0.14)	0.85		0.94
		2 years	-0.21 (1.11) [285]	-0.19 (0.95) [283]	0.04 (-0.13 to 0.20)	0.67	0.42	
		3 years	-0.34 (1.07) [310]	-0.28 (1.00) [326]	-0.03 (-0.19 to 0.13)	0.69		
	Girls	1 year	-0.15 (1.09) [360]	-0.04 (1.07) [355]	-0.10 (-0.25 to 0.06)	0.21		
		2 years	-0.08 (0.99) [328]	0.05 (1.06) [323]	-0.12 (-0.28 to 0.04)	0.14	0.34	
		3 years	-0.19 (1.02) [360]	-0.02 (1.08) [365]	-0.13 (-0.28 to 0.03)	0.11		
By calcium intake[2]	<median	1 year	-0.16 (1.05) [314]	-0.10 (1.02) [331]	-0.04 (-0.18 to 0.11)	0.64		0.07
		2 years	-0.17 (0.96) [288]	-0.05 (0.96) [310]	-0.04 (-0.19 to 0.11)	0.64	0.02	
		3 years	-0.28 (0.99) [312]	-0.15 (0.99) [362]	-0.08 (-0.23 to 0.07)	0.29		
	≥median	1 year	-0.25 (1.16) [327]	-0.18 (1.13) [314]	-0.05 (-0.21 to 0.12)	0.60		
		2 years	-0.07 (1.12) [307]	-0.06 (1.06) [279]	-0.03 (-0.20 to 0.14)	0.76	0.72	
		3 years	-0.20 (1.09) [337]	-0.13 (1.11) [311]	-0.06 (-0.22 to 0.11)	0.50		
By baseline 25(OH)D concentration[3]	<75 nmol/L	1 year	-0.23 (1.13) [349]	-0.03 (1.11) [332]	-0.17 (-0.33 to -0.01)	0.04		0.50
		2 years	-0.14 (1.11) [325]	0.05 (1.06) [323]	-0.12 (-0.28 to 0.04)	0.15	0.53	
		3 years	-0.25 (1.10) [340]	-0.06 (1.07) [359]	-0.15 (-0.31 to 0.01)	0.07		
	≥75 nmol/L	1 year	-0.22 (1.15) [187]	-0.25 (0.98) [201]	0.02 (-0.17 to 0.22)	0.82		
		2 years	-0.17 (1.00) [171]	-0.08 (0.96) [179]	-0.07 (-0.26 to 0.13)	0.50	0.15	
		3 years	-0.30 (1.00) [196]	-0.18 (0.99) [206]	-0.06 (-0.25 to 0.13)	0.53		

Abbreviations: 25(OH)D, 25-hydroxyvitamin D. CI, confidence interval. S.d., standard deviation. n, number.

Footnotes. 1, adjusted for baseline value and school of attendance. **2,** median calcium intake 466 mg/day. **3,** deseasonalised values.

Confidential: For Review Only

Table 3. Mean BMI-for-age z-scores at annual follow-up by allocation, main study participants: overall and by sub-group

			Vitamin D arm: mean value (s.d.) [n]	Placebo arm: mean value (s.d.) [n]	Adjusted mean difference (95% CI)[1]	P for timepoint	Overall P	P for interaction
Overall		1 year	0.16 (1.03) [664]	0.17 (1.02) [661]	-0.02 (-0.14 to 0.09)	0.68	0.86	--
		2 years	0.15 (1.41) [613]	0.25 (1.21) [606]	-0.09 (-0.20 to 0.03)	0.14		
		3 years	0.17 (1.07) [669]	0.21 (1.17) [691]	-0.04 (-0.16 to 0.07)	0.45		
By sex	Boys	1 year	0.08 (0.99) [304]	-0.00 (0.99) [306]	0.09 (-0.08 to 0.27)	0.30	0.59	0.29
		2 years	0.00 (1.68) [285]	-0.04 (1.30) [283]	0.04 (-0.14 to 0.22)	0.64		
		3 years	-0.02 (1.04) [309]	-0.12 (1.20) [326]	0.09 (-0.09 to 0.26)	0.33		
	Girls	1 year	0.23 (1.06) [360]	0.32 (1.02) [355]	-0.14 (-0.28 to 0.00)	0.06	0.26	
		2 years	0.28 (1.10) [328]	0.50 (1.06) [323]	-0.21 (-0.36 to -0.06)	0.00		
		3 years	0.33 (1.06) [360]	0.50 (1.07) [365]	-0.17 (-0.32 to -0.03)	0.02		
By calcium intake[2]	<median	1 year	0.15 (1.01) [314]	0.15 (1.03) [331]	-0.01 (-0.17 to 0.14)	0.86	0.16	0.08
		2 years	0.18 (1.08) [288]	0.21 (1.34) [310]	-0.03 (-0.19 to 0.13)	0.75		
		3 years	0.21 (1.07) [312]	0.20 (1.14) [362]	0.01 (-0.14 to 0.17)	0.87		
	≥median	1 year	0.18 (1.04) [327]	0.22 (1.01) [314]	-0.04 (-0.21 to 0.13)	0.61	0.27	
		2 years	0.13 (1.67) [307]	0.31 (1.07) [279]	-0.15 (-0.32 to 0.03)	0.10		
		3 years	0.15 (1.07) [336]	0.22 (1.23) [311]	-0.09 (-0.26 to 0.08)	0.31		
By baseline 25(OH)D concentration[3]	<75 nmol/L	1 year	0.30 (1.03) [349]	0.28 (0.98) [332]	0.03 (-0.14 to 0.19)	0.76	0.31	0.31
		2 years	0.22 (1.64) [325]	0.31 (1.33) [323]	-0.07 (-0.24 to 0.10)	0.41		
		3 years	0.25 (1.07) [340]	0.34 (1.08) [359]	-0.09 (-0.25 to 0.08)	0.31		
	≥75 nmol/L	1 year	0.02 (0.97) [187]	0.09 (1.02) [201]	-0.06 (-0.25 to 0.13)	0.57	0.58	
		2 years	0.01 (1.05) [171]	0.18 (1.00) [179]	-0.11 (-0.30 to 0.09)	0.28		
		3 years	0.09 (1.00) [196]	0.06 (1.17) [206]	0.02 (-0.17 to 0.21)	0.87		

Abbreviations: 25(OH)D, 25-hydroxyvitamin D. CI, confidence interval. S.d., standard deviation. n, number.
Footnotes. 1, adjusted for baseline value and school of attendance. **2,** median calcium intake 466 mg/day. **3,** deseasonalised values.

Confidential: For Review Only

Table 4. End-study pubertal development indices by allocation, sub-study participants: overall and by sub-group

				Vitamin D arm: mean value (s.d.) [n]	Placebo arm: mean value (s.d.) [n]	Adjusted mean difference (95% CI)[1]	P	P for interaction
Males	Pubic hair, mean Tanner score (s.d.) [n]	Overall		3.15 (0.91) [96]	2.81 (0.99) [90]	0.25 (-0.02 to 0.51)	0.07	--
		Calcium intake[2]	<median	3.13 (0.93) [46]	2.73 (1.14) [52]	0.29 (-0.10 to 0.67)	0.14	0.39
			≥median	3.15 (0.90) [48]	2.92 (0.75) [38]	0.16 (-0.19 to 0.52)	0.37	
		Baseline 25(OH)D concentration[3]	<75 nmol/L	3.11 (0.92) [44]	2.88 (0.92) [42]	0.07 (-0.28 to 0.41)	0.70	0.39
			≥75 nmol/L	3.18 (0.94) [34]	2.70 (1.18) [30]	0.43 (-0.07 to 0.93)	0.09	
	External genitalia, mean Tanner score (s.d.) [n]	Overall		3.19 (0.91) [96]	2.90 (0.99) [90]	0.17 (-0.09 to 0.43)	0.19	--
		Calcium intake[2]	<median	3.28 (0.89) [46]	2.81 (1.10) [52]	0.35 (-0.01 to 0.71)	0.06	0.07
			≥median	3.10 (0.93) [48]	3.03 (0.82) [38]	-0.04 (-0.40 to 0.33)	0.84	
		Baseline 25(OH)D concentration[3]	<75 nmol/L	3.27 (0.82) [44]	2.90 (0.91) [42]	0.22 (-0.10 to 0.54)	0.18	0.81
			≥75 nmol/L	3.15 (1.02) [34]	2.87 (1.17) [30]	0.30 (-0.17 to 0.77)	0.21	
Females	Pubic hair, mean Tanner score (s.d.) [n]	Overall		3.10 (0.98) [105]	3.11 (0.98) [99]	-0.09 (-0.35 to 0.17)	0.50	--
		Calcium intake[2]	<median	3.15 (0.98) [47]	3.15 (1.01) [46]	0.01 (-0.38 to 0.40)	0.96	0.60
			≥median	3.11 (0.92) [54]	3.10 (0.97) [50]	-0.23 (-0.56 to 0.10)	0.17	
		Baseline 25(OH)D concentration[3]	<75 nmol/L	3.14 (0.90) [59]	3.13 (0.88) [55]	-0.03 (-0.35 to 0.28)	0.83	0.94
			≥75 nmol/L	2.96 (1.22) [26]	3.04 (1.08) [24]	-0.14 (-0.74 to 0.46)	0.65	
	Breast development, mean Tanner score (s.d.) [n]	Overall		3.15 (0.87) [105]	3.10 (0.91) [99]	-0.03 (-0.26 to 0.20)	0.80	--
		Calcium intake[2]	<median	3.15 (0.91) [47]	3.22 (0.92) [46]	-0.06 (-0.41 to 0.29)	0.74	0.93
			≥median	3.20 (0.81) [54]	3.04 (0.90) [50]	-0.05 (-0.36 to 0.26)	0.76	
		Baseline 25(OH)D concentration[3]	<75 nmol/L	3.27 (0.85) [59]	3.07 (0.86) [55]	0.15 (-0.16 to 0.45)	0.34	0.48
			≥75 nmol/L	3.08 (0.93) [26]	3.08 (0.97) [24]	-0.08 (-0.55 to 0.38)	0.73	
	Proportion menstruating	Overall		67/105 (63.81)	60/99 (60.61)	0.95 (0.51 to 1.77)	0.86	--
		Calcium intake[2]	<median	28/47 (59.57)	26/46 (56.52)	1.33 (0.50 to 3.56)	0.57	0.24
			≥median	38/54 (70.37)	34/50 (68.00)	0.56 (0.21 to 1.52)	0.26	
		Baseline 25(OH)D concentration[3]	<75 nmol/L	43/59 (72.88)	35/55 (63.64)	1.39 (0.59 to 3.28)	0.45	0.59
			≥75 nmol/L	16/26 (61.54)	15/24 (62.50)	0.90 (0.26 to 3.15)	0.87	
	Mean age at menarche, years (s.d.) [n]	Overall		11.94 (0.93) [66]	11.86 (0.66) [58]	-0.03 (-0.31 to 0.25)	0.84	--
		Calcium intake[2]	<median	11.89 (0.83) [28]	11.71 (0.69) [24]	0.12 (-0.26 to 0.49)	0.54	0.32
			≥median	11.97 (1.01) [37]	11.97 (0.63) [34]	-0.11 (-0.51 to 0.29)	0.58	
		Baseline 25(OH)D concentration[3]	<75 nmol/L	12.00 (0.88) [42]	11.88 (0.69) [34]	0.04 (-0.31 to 0.39)	0.82	0.64
			≥75 nmol/L	11.88 (1.15) [16]	11.93 (0.62) [14]	-0.14 (-0.73 to 0.44)	0.63	

Abbreviations: 25(OH)D, 25-hydroxyvitamin D. CI, confidence interval. S.d., standard deviation. n, number.
Footnotes. 1, adjusted for school of attendance. **2,** median calcium intake 466 mg/day. **3,** deseasonalised values.

Confidential: For Review Only

Figure 1. Participant Flow Diagram

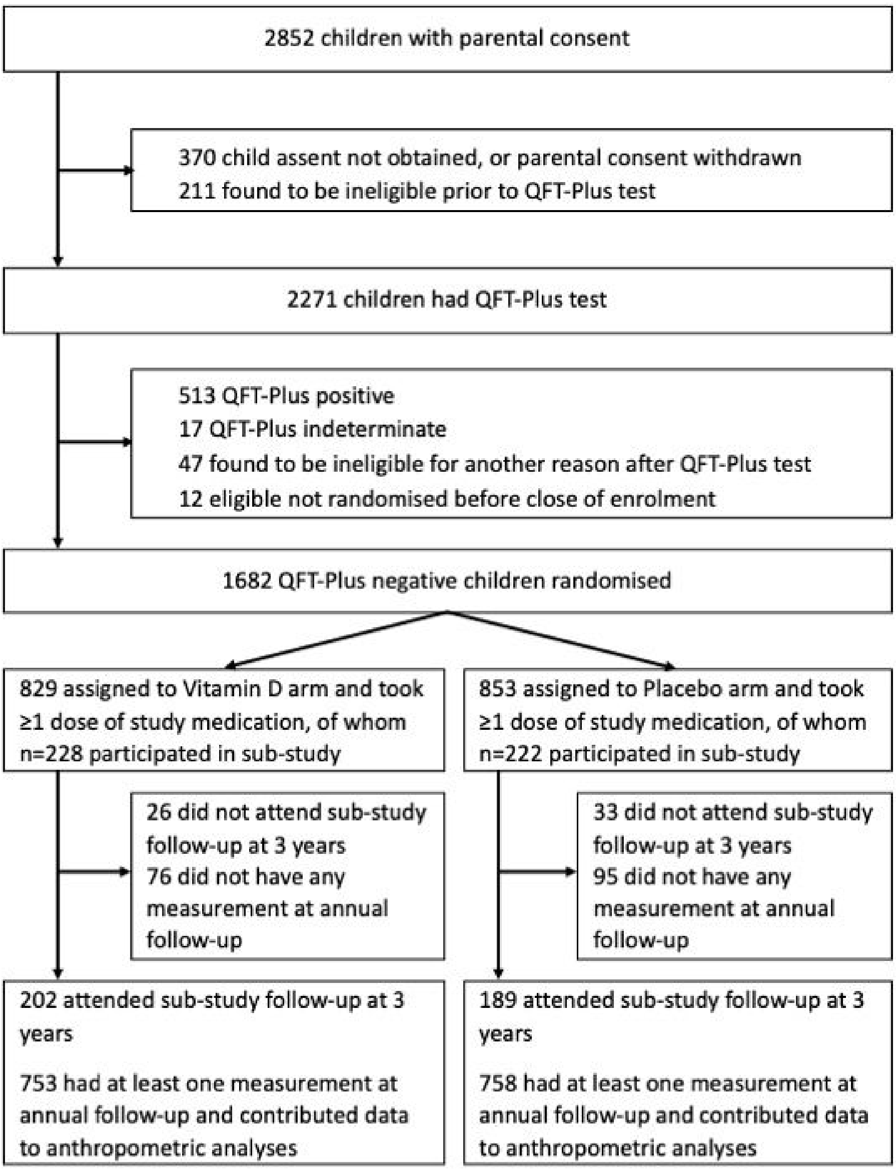

References

1. Eccles JS. The development of children ages 6 to 14. *Future Child* 1999; **9**(2): 30-44.
2. Dewey KG, Begum K. Long-term consequences of stunting in early life. *Maternal & child nutrition* 2011; **7 Suppl 3**(Suppl 3): 5-18.
3. Vaivada T, Akseer N, Akseer S, Somaskandan A, Stefopulos M, Bhutta ZA. Stunting in childhood: an overview of global burden, trends, determinants, and drivers of decline. *Am J Clin Nutr* 2020; **112**(Suppl 2): 777S-91S.
4. Di Cesare M, Soric M, Bovet P, et al. The epidemiological burden of obesity in childhood: a worldwide epidemic requiring urgent action. *BMC medicine* 2019; **17**(1): 212.
5. Eckert-Lind C, Busch AS, Petersen JH, et al. Worldwide Secular Trends in Age at Pubertal Onset Assessed by Breast Development Among Girls: A Systematic Review and Meta-analysis. *JAMA pediatrics* 2020; **174**(4): e195881.
6. Bikle DD. Vitamin D: Production, Metabolism and Mechanisms of Action. In: Feingold KR, Anawalt B, Blackman MR, et al., eds. Endotext. South Dartmouth (MA); 2000.
7. Gilbert-Diamond D, Baylin A, Mora-Plazas M, et al. Vitamin D deficiency and anthropometric indicators of adiposity in school-age children: a prospective study. *Am J Clin Nutr* 2010; **92**(6): 1446-51.
8. Foo LH, Zhang Q, Zhu K, et al. Relationship between vitamin D status, body composition and physical exercise of adolescent girls in Beijing. *Osteoporos Int* 2009; **20**(3): 417-25.
9. Zakharova I, Klimov L, Kuryaninova V, et al. Vitamin D Insufficiency in Overweight and Obese Children and Adolescents. *Front Endocrinol (Lausanne)* 2019; **10**: 103.
10. Plesner JL, Dahl M, Fonvig CE, et al. Obesity is associated with vitamin D deficiency in Danish children and adolescents. *Journal of pediatric endocrinology & metabolism : JPEM* 2018; **31**(1): 53-61.
11. Theodoratou E, Tzoulaki I, Zgaga L, Ioannidis JP. Vitamin D and multiple health outcomes: umbrella review of systematic reviews and meta-analyses of observational studies and randomised trials. *BMJ* 2014; **348**: g2035.
12. Lee HS, Kim YJ, Shim YS, Jeong HR, Kwon E, Hwang JS. Associations between serum vitamin D levels and precocious puberty in girls. *Annals of pediatric endocrinology & metabolism* 2014; **19**(2): 91-5.
13. Liu S, Zhu X, Wang Y, Yan S, Li D, Cui W. The association between vitamin D levels and precocious puberty: a meta-analysis. *Journal of pediatric endocrinology & metabolism : JPEM* 2020; **33**(3): 427-9.
14. Gou Z, Li F, Qiao F, Maimaititusvn G, Liu F. Causal associations between insulin-like growth factor 1 and vitamin D levels: a two-sample bidirectional Mendelian randomization study. *Front Nutr* 2023; **10**: 1162442.
15. Nimitphong H, Park E, Lee MJ. Vitamin D regulation of adipogenesis and adipose tissue functions. *Nutr Res Pract* 2020; **14**(6): 553-67.
16. Ganmaa D, Stuart JJ, Sumberzul N, et al. Vitamin D supplementation and growth in urban Mongol school children: Results from two randomized clinical trials. *PLoS One* 2017; **12**(5): e0175237.
17. Ganmaa D, Bromage S, Khudyakov P, Erdenenbaatar S, Delgererekh B, Martineau AR. Influence of Vitamin D Supplementation on Growth, Body Composition, and Pubertal Development Among School-aged Children in an Area With a High Prevalence of Vitamin D Deficiency: A Randomized Clinical Trial. *JAMA pediatrics* 2023; **177**(1): 32-41.
18. Middelkoop K, Stewart J, Walker N, et al. Vitamin D Supplementation to Prevent Tuberculosis Infection in South African Schoolchildren: Multicentre Phase 3 Double-Blind Randomised Placebo-Controlled Trial (ViDiKids). *Int J Infect Dis* 2023; **134**: 63-70.

Confidential: For Review Only

19. Middelkoop K, Micklesfield LK, Walker N, et al. Influence of vitamin D supplementation on bone mineral content, bone turnover markers and fracture risk in South African schoolchildren: multicentre double-blind randomised placebo-controlled trial (ViDiKids). *J Bone Miner Res* 2023; **in press 2023**.
20. Graham BL, Steenbruggen I, Miller MR, et al. Standardization of Spirometry 2019 Update. An Official American Thoracic Society and European Respiratory Society Technical Statement. *Am J Respir Crit Care Med* 2019; **200**(8): e70-e88.
21. Chavarro JE, Watkins DJ, Afeiche MC, et al. Validity of Self-Assessed Sexual Maturation Against Physician Assessments and Hormone Levels. *J Pediatr* 2017; **186**: 172-8 e3.
22. Middelkoop K, Walker N, Stewart J, Delport C, Martineau AR. Prevalence and Determinants of Vitamin D Deficiency in 1825 Cape Town Primary Schoolchildren: A Cross-Sectional Study. *Nutrients* 2022; **14**(6): 1263.
23. de Onis M, Onyango AW, Borghi E, Siyam A, Nishida C, Siekmann J. Development of a WHO growth reference for school-aged children and adolescents. *Bulletin of the World Health Organization* 2007; **85**(9): 660-7.
24. Ganmaa D, Khudyakov P, Buyanjargal U, et al. Prevalence and Determinants of QuantiFERON-Diagnosed Tuberculosis Infection in 9810 Mongolian Schoolchildren. *Clin Infect Dis* 2019; **69**(5): 813-9.
25. Norris SA, Richter LM. Usefulness and Reliability of Tanner Pubertal Self-Rating to Urban Black Adolescents in South Africa. *Journal of Research on Adolescence* 2005; **15**: 609-24.

Confidential: For Review Only

Influence of vitamin D supplementation on growth, body composition, pubertal development and spirometry in South African schoolchildren: a randomised controlled trial (ViDiKids)

Supplemental Material

Confidential: For Review Only

Contents

Supplementary Methods 2
Enrolment 2
Randomisation and blinding 2
Intervention 4
Laboratory Assessments 5
Statistical Analyses 5
Table S1. Mean waist circumference-for-age z-scores at annual follow-up by allocation, main trial participants: overall and by sub-group 7
Table S2. Mean waist-to-height ratio z-scores at annual follow-up by allocation, main trial participants: overall and by sub-group 8
Table S3. Fat mass and fat-free soft tissue mass at 3-year follow-up by allocation, sub-study participants: overall and by sub-group 9
Table S4. Spirometric outcomes at 3-year follow-up by allocation, sub-study participants: overall and by sub-group 10

Supplementary Methods

Enrolment

Parents or legal guardians were invited to provide written informed consent for their child to participate in the main trial during a home visit, unless their child was eligible for the sub-study, in which case they were invited to provide written informed consent for their child to participate in both the main trial and the sub-study until a total of 450 sub-study participants were randomised. If parents / legal guardians consented, they were asked to provide details of their child's dietary intake of foods containing vitamin D and calcium in the previous month, which were captured on an electronic case report form as previously reported.[19] Their children were then invited to provide written assent to participate in the main trial +/- the sub-study (if eligible) at a school-based visit. If they agreed, a clinically trained member of the study team screened them for symptoms and signs of rickets. For all participants, a blood sample was taken for a QFT-Plus assay and separation and storage of serum for determination of 25(OH)D concentrations as described below. Participants were reviewed when baseline QFT-Plus results were available. Those with a positive QFT-Plus result were excluded from the trial and screened for active TB. Those with an indeterminate QFT-Plus result were excluded from the trial without screening for active TB. Those with a negative QFT-Plus result were deemed eligible to participate and underwent measurement of weight (using a digital floor scale, Charder Medical, Taichung City, Taiwan), height (using a portable HM200P stadiometer, Charder Medical) and waist circumference (using a measuring tape). Sub-study participants also underwent spirometry according to ERS/ATS standards[20] using a portable spirometer (Carefusion, San Diego, CA) and measurement of body composition by dual energy x-ray absorptiometry (DXA) as described below.

Randomisation and blinding

Eligible participants were individually randomised to receive a weekly capsule containing vitamin D_3 or placebo for three years, with a one-to-one allocation ratio. Randomisation was stratified by school of attendance (a potential predictor of risk of QFT conversion) as follows. Prior to the start of recruitment, Mrs Claire Chan

Confidential: For Review Only

(Independent statistician, Pragmatic Clinical Trials Unit, Queen Mary University of London) prepared two school randomisation lists, each comprising 20 pairs of 2-letter randomisation codes, with each pair allocated to a single school identifier (e.g. School 1 was allocated codes 'AC' and 'MO', School 2 was allocated codes 'BD' and 'NP'). One 2-letter randomisation code within each pair was then randomly assigned to the vitamin D arm of the trial, and the other was assigned to the placebo arm, using a computer-generated random sequence (e.g. 'AC' and 'BD' were assigned to 'vitamin D', 'MO' and 'NP' were assigned to placebo). Mrs Chan also prepared 40 separate participant randomisation lists (one for each potential participating school). Each of these participant randomisation lists comprised 999 5-digit numbers, each consisting of a 2-digit school identifier from 01 to 25 that was constant for, and unique to, each list, followed by a 3-digit participant identifier from 001 to 999 (e.g. 01-001, 01-002, 02-001, 02-002). These 5-digit numbers were then randomly assigned to one or other of the 2-letter randomisation codes allocated to that school in blocks of ten, using a computer-generated random sequence (e.g. for School 1, sequence was 01-001-MO, 01-002-AC, etc.; for School 2, sequence was 02-001-BD, 02-002-BD, etc.).

Active and placebo capsules were shipped from Tishcon Corporation (Westbury, NY, USA) to Lekoko Pharmaceutical Management Consultancy (Johannesburg, South Africa) in boxes that were labelled 'vitamin D' or 'placebo' according to their contents. On arrival, these capsules were packed into bottles, each containing either 150 vitamin D capsules or 150 placebo capsules. The school randomisation list was then used to label these bottles with 2-letter randomisation codes according to their contents (i.e. bottles containing vitamin D capsules were labelled with 'AC', 'BD' or another 2-letter code assigned to the vitamin D arm of the trial, while bottles containing placebo capsules were labelled 'MO', 'NP' or another 2-letter code assigned to the placebo arm of the trial) until sufficient capsules for 23 schools had been bottled and labelled. Bottling and labelling was performed under the supervision of Mr Bobby Hamman (Lekoko PMC), with independent monitoring performed by staff from OnQ Contract Research Organisation; none of these individuals was involved with data collection. Children screened at each school were assigned consecutive 5-digit numbers at enrolment by study field workers, and if they were subsequently found to be eligible for randomisation (i.e. if their baseline QFT-Plus result was negative) then the participant randomisation list for their school of attendance was used to determine

https://mc.manuscriptcentral.com/bmjpo

Confidential: For Review Only

their allocation, i.e. they received study medication from bottles labelled with the 2-letter code linked to their 5-digit ID in the participant randomization list for the duration of the trial. For example, participant 01-001 would receive study medication labelled 'MO' (i.e. placebo), 01-002 would receive medication labelled 'AC' (i.e. vitamin D), 02-001 would receive medication labelled 'BD' (vitamin D), and 02-002 would receive medication labelled 'BD' (vitamin D) throughout the trial. Copies of the school randomisation list were held by Mrs Chan and by members of the DSMB. Neither participants nor trial staff had access to it, and treatment allocation was concealed from participants, care providers and all trial staff (including senior investigators and those assessing outcomes) so that the double-blind was maintained. The school randomisation lists were made available to Dr Neil Walker (trial statistician) following completion of the trial, who used them to un-blind allocation and to analyse the trial: he was not therefore masked to group assignment during statistical analysis.

Intervention

Study medication comprised a 3-year course of weekly soft gel capsules manufactured by the Tishcon Corporation (Westbury, NY, USA), containing either 0.25 mg (10,000 international units) cholecalciferol (vitamin D_3) in olive oil (intervention arm) or olive oil without any vitamin D_3 content (placebo arm). Active and placebo capsules had identical appearance and taste. Capsules were taken under direct observation of study staff during school term time. Further details of administration of study medication are provided in Supplementary Material. During summer holidays (8 weeks), packs containing 8 doses of study medication were provided for administration by parents, together with a participant diary. Following shorter school holidays (≤4 weeks), and/or if participants missed one or more doses of study medication during term time, up to 4 'catch-up' doses were administered at the first weekly visit attended following the missed dose(s). During the initial national lockdown for COVID-19 in South Africa (27th March to 1st May 2020), participants did not receive any study medication. During subsequent school closures due to COVID-19, two rounds of 8-week holiday packs were provided to participants, which were sufficient to cover their requirements until schools re-opened.

Confidential: For Review Only

Laboratory Assessments

Biochemical analyses were performed at the Bioanalytical Facility, University of East Anglia (Norwich, UK) according to manufacturers' instructions and under Good Clinical and Laboratory Practice conditions. Serum concentrations of 25(OH)D_3 were measured using liquid chromatography tandem mass spectrometry (LC-MS/MS) as previously described.[22] 25(OH)D_3 was calibrated using standard reference material SRM972a from the National Institute of Science and Technology (NIST), and the assay showed linearity between 0 and 200 nmol/L. The inter/intra-assay coefficient of variation (CV) across the assay range was ≤9%, and the lower limit of quantification was 0.1 nmol/L. The assay showed <6% accuracy bias against NIST reference method on the vitamin D external quality assessment (DEQAS) scheme (http://www.deqas.org/; accessed on 30th November 2022). QFT-Plus assays were performed by the Bio Analytical Research Corporation South Africa (Johannesburg, South Africa) according to the manufacturer's instructions.

Statistical Analyses

Statistical analyses were performed using Stata software (Version 17.0; StataCorp, College Station, Texas, United States) according to intention to treat. Anthropometric measurements were used to compute age- and sex-adjusted z-scores for height, BMI, waist circumference and waist-to-height ratio, using the WHO 2007 Lambda, Mu and Sigma method.[23] Effects of treatment on the above Z-transformed outcomes were estimated by fitting allocation to vitamin D vs. placebo as the sole fixed effect in a mixed effects linear regression model with a random effect for repeated assessments of each individual participant (at baseline and years 1, 2 and 3 of follow-up) and a random effect of school of attendance, with results reported as treatment differences with 95% confidence intervals. Effects of treatment on DXA and spirometric outcomes measured at 3-year follow-up were analysed in each case using a multi-level mixed model with an adjustment for analogous baseline score and a random effect for school. Tanner metrics on pubertal development were analysed in a similar fashion, but

Confidential: For Review Only

restricted to the applicable sex and without baseline adjustment. Binary outcomes measured at 3-year follow-up only were analysed using a mixed-effects logistic regression model, with treatment allocation as the sole fixed effect and school attended as a random intercept. Pre-specified sub-group analyses were conducted to determine whether the effect of vitamin D supplementation was modified by sex (male vs. female), baseline deseasonalised 25(OH)D_3 concentration (<75 vs. ≥75 nmol/L, calculated using a sinusoidal model as previously described)[24] and calcium intake (< vs. ≥ median value of 466 mg/day, calculated from a dietary intake questionnaire as previously described).[19] These were performed by repeating efficacy analyses with the inclusion of an interaction term between allocation (to vitamin D vs. placebo) and each posited effect-modifier with presentation of the P-value associated with this interaction term. Effect of sex as a subgroup was not considered in relation to analyses of Tanner outcomes, as these are sex-specific. Given the number of potential effect modifiers and secondary outcome measures these analyses are considered exploratory. Interim safety assessments, where Independent Data Monitoring Committee (IDMC) members reviewed accumulating serious adverse event data, were performed at 6-monthly intervals. At each review, the IDMC recommended continuation of the trial. No interim efficacy analysis was performed.

Confidential: For Review Only

Table S1. Mean waist circumference-for-age z-scores at annual follow-up by allocation, main trial participants: overall and by sub-group

			Vitamin D: mean value (s.d.) [n]	Placebo: mean value (s.d.) [n]	Adjusted mean difference (95% CI)[1]	P for timepoint	P for trend	P for interaction (treatment*subgroup)
Overall		1 year	-0.03 (0.89) [664]	-0.00 (0.87) [660]	-0.03 (-0.12 to 0.06)	0.47	0.01	
		2 years	0.19 (0.87) [612]	0.24 (0.87) [608]	-0.06 (-0.15 to 0.04)	0.24		
		3 years	0.15 (0.83) [667]	0.21 (0.88) [686]	-0.07 (-0.16 to 0.02)	0.12		
By sex	Boys	1 year	-0.14 (0.84) [304]	-0.20 (0.88) [305]	0.06 (-0.07 to 0.19)	0.39	0.07	0.93
		2 years	0.11 (0.84) [286]	0.06 (0.88) [284]	0.05 (-0.08 to 0.18)	0.47		
		3 years	0.03 (0.83) [309]	0.03 (0.85) [325]	-0.02 (-0.15 to 0.11)	0.78		
	Girls	1 year	0.06 (0.92) [360]	0.17 (0.82) [355]	-0.12 (-0.24 to 0.00)	0.05	0.04	
		2 years	0.26 (0.88) [326]	0.41 (0.84) [324]	-0.15 (-0.28 to -0.03)	0.01		
		3 years	0.25 (0.82) [358]	0.37 (0.86) [361]	-0.13 (-0.25 to -0.01)	0.04		
By calcium intake[2]	<median	1 year	-0.04 (0.89) [314]	-0.05 (0.86) [331]	0.00 (-0.12 to 0.13)	0.96	0.02	0.64
		2 years	0.19 (0.86) [289]	0.19 (0.89) [312]	0.00 (-0.12 to 0.13)	0.94		
		3 years	0.16 (0.81) [310]	0.21 (0.85) [358]	-0.03 (-0.16 to 0.09)	0.62		
	≥median	1 year	-0.01 (0.89) [327]	0.06 (0.89) [313]	-0.07 (-0.20 to 0.06)	0.28	0.10	
		2 years	0.20 (0.87) [305]	0.30 (0.86) [279]	-0.10 (-0.23 to 0.03)	0.14		
		3 years	0.14 (0.87) [337]	0.22 (0.91) [310]	-0.11 (-0.24 to 0.02)	0.10		
By baseline 25(OH)D concentration[3]	<75 nmol/L	1 year	0.09 (0.90) [349]	0.08 (0.87) [332]	0.02 (-0.10 to 0.15)	0.72	0.08	0.68
		2 years	0.26 (0.89) [324]	0.31 (0.88) [324]	-0.02 (-0.15 to 0.11)	0.75		
		3 years	0.21 (0.85) [338]	0.29 (0.85) [356]	-0.06 (-0.18 to 0.06)	0.34		
	≥75 nmol/L	1 year	-0.19 (0.84) [187]	-0.06 (0.85) [200]	-0.14 (-0.30 to 0.02)	0.08	0.07	
		2 years	0.05 (0.81) [171]	0.20 (0.82) [179]	-0.15 (-0.31 to 0.01)	0.07		
		3 years	0.03 (0.76) [196]	0.14 (0.87) [205]	-0.11 (-0.26 to 0.05)	0.19		

Abbreviations: 25(OH)D, 25-hydroxyvitamin D. CI, confidence interval. S.d., standard deviation. n, number.
Footnotes. 1, adjusted for baseline value and school of attendance. **2,** median calcium intake 466 mg/day. **3,** deseasonalised values.

Confidential: For Review Only

Table S2. Mean waist-to-height ratio z-scores at annual follow-up by allocation, main trial participants: overall and by sub-group

			**Vitamin D: mean value (s.d.) [n]**	**Placebo: mean value (s.d.) [n]**	**Adjusted mean difference (95% CI)[1]**	**P for timepoint**	**P for trend**	**P for interaction (treatment*subgroup)**
Overall		1 year	-0.01 (0.82) [664]	-0.01 (0.81) [659]	-0.02 (-0.10 to 0.07)	0.67		
		2 years	0.15 (0.83) [610]	0.19 (0.83) [606]	-0.04 (-0.13 to 0.04)	0.32	0.01	
		3 years	0.11 (0.80) [667]	0.15 (0.83) [686]	-0.05 (-0.14 to 0.03)	0.22		
By sex	Boys	1 year	-0.04 (0.76) [304]	-0.12 (0.80) [304]	0.06 (-0.07 to 0.18)	0.36		
		2 years	0.17 (0.76) [284]	0.12 (0.78) [283]	0.03 (-0.09 to 0.16)	0.60	0.08	
		3 years	0.09 (0.78) [309]	0.08 (0.78) [325]	-0.01 (-0.13 to 0.11)	0.85		
	Girls	1 year	0.02 (0.87) [360]	0.09 (0.80) [355]	-0.09 (-0.21 to 0.03)	0.14		
		2 years	0.15 (0.88) [326]	0.26 (0.86) [323]	-0.12 (-0.24 to 0.00)	0.06	0.08	0.83
		3 years	0.13 (0.82) [358]	0.21 (0.87) [361]	-0.10 (-0.21 to 0.02)	0.11		
By calcium intake[2]	<median	1 year	-0.05 (0.81) [314]	-0.09 (0.82) [331]	0.02 (-0.10 to 0.14)	0.71		
		2 years	0.17 (0.79) [288]	0.13 (0.84) [310]	0.02 (-0.11 to 0.14)	0.77	0.03	
		3 years	0.13 (0.78) [310]	0.15 (0.80) [358]	-0.02 (-0.14 to 0.10)	0.74		
	≥median	1 year	0.01 (0.85) [327]	0.08 (0.80) [312]	-0.08 (-0.20 to 0.05)	0.22		
		2 years	0.15 (0.85) [304]	0.25 (0.82) [279]	-0.10 (-0.23 to 0.03)	0.12	0.15	0.64
		3 years	0.09 (0.83) [337]	0.15 (0.88) [310]	-0.09 (-0.21 to 0.04)	0.16		
By baseline 25(OH)D concentration[3]	<75 nmol/L	1 year	0.11 (0.82) [349]	0.04 (0.80) [331]	0.08 (-0.04 to 0.19)	0.22		
		2 years	0.23 (0.84) [323]	0.21 (0.83) [323]	0.03 (-0.09 to 0.15)	0.66	0.07	
		3 years	0.17 (0.82) [338]	0.19 (0.80) [356]	-0.02 (-0.14 to 0.10)	0.78		
	≥75 nmol/L	1 year	-0.17 (0.80) [187]	-0.04 (0.83) [200]	-0.15 (-0.44 to 0.15)	0.33	0.15	
		2 years	0.02 (0.76) [170]	0.16 (0.77) [179]	-0.15 (-0.46 to 0.16)	0.34		0.88
		3 years	0.01 (0.75) [196]	0.09 (0.81) [205]	-0.10 (-0.39 to 0.19)	0.51		

Abbreviations: 25(OH)D, 25-hydroxyvitamin D. CI, confidence interval. S.d., standard deviation. n, number.
Footnotes. 1, adjusted for baseline value and school of attendance. **2,** median calcium intake 466 mg/day. **3,** deseasonalised values.

Confidential: For Review Only

Table S3. Fat mass and fat-free soft tissue mass at 3-year follow-up by allocation, sub-study participants: overall and by sub-group

		Vitamin D: mean value (s.d.) [n]	Placebo: mean value (s.d.) [n]	Adjusted mean difference (95% CI)[1]	P	P for interaction
**Fat mass, kg**						
Overall		6.04 (3.86) [202]	6.28 (3.99) [189]	-0.15 (-0.51 to 0.20)	0.40	
By sex	Boys	4.80 (3.11) [97]	4.78 (3.05) [89]	0.18 (-0.30 to 0.66)	0.45	0.14
	Girls	7.19 (4.13) [105]	7.61 (4.27) [100]	-0.45 (-0.96 to 0.05)	0.08	
By calcium intake[2]	<median	5.73 (3.32) [94]	5.74 (3.55) [97]	0.04 (-0.42 to 0.49)	0.88	0.34
	≥median	6.41 (4.35) [102]	6.88 (4.41) [89]	-0.34 (-0.90 to 0.23)	0.24	
By baseline 25(OH)D concentration[3]	<75 nmol/L	6.74 (4.28) [103]	6.64 (4.23) [96]	-0.32 (-0.85 to 0.21)	0.24	0.54
	≥75 nmol/L	5.39 (3.47) [60]	5.85 (3.71) [54]	-0.04 (-0.62 to 0.55)	0.91	
By baseline fat mass	<median	3.72 (1.08) [103]	3.65 (1.20) [90]	0.02 (-0.24 to 0.27)	0.88	0.42
	≥median	8.46 (4.20) [99]	8.67 (4.15) [99]	-0.27 (-0.91 to 0.37)	0.41	
**Fat-free soft tissue mass, kg**						
Overall		13.22 (2.87) [202]	13.07 (2.70) [189]	-0.13 (-0.42 to 0.15)	0.36	
By sex	Boys	14.00 (2.87) [97]	13.54 (3.03) [89]	-0.01 (-0.43 to 0.41)	0.96	0.08
	Girls	12.50 (2.69) [105]	12.64 (2.29) [100]	-0.28 (-0.63 to 0.06)	0.10	
By calcium intake[2]	<median	13.31 (2.68) [94]	13.08 (2.67) [97]	-0.09 (-0.48 to 0.30)	0.67	0.68
	≥median	13.23 (3.07) [102]	13.16 (2.69) [89]	-0.16 (-0.59 to 0.27)	0.48	
By baseline 25(OH)D concentration[3]	<75 nmol/L	13.33 (3.06) [103]	13.33 (2.79) [96]	-0.21 (-0.62 to 0.21)	0.33	0.91
	≥75 nmol/L	13.30 (3.03) [60]	13.01 (2.72) [54]	-0.17 (-0.68 to 0.34)	0.51	
By baseline fat-free soft tissue mass	<median	11.06 (1.54) [89]	11.40 (1.53) [103]	-0.13 (-0.43 to 0.16)	0.38	0.95
	≥median	14.92 (2.52) [113]	15.06 (2.43) [86]	-0.14 (-0.60 to 0.33)	0.57	

Abbreviations: 25(OH)D, 25-hydroxyvitamin D. CI, confidence interval. S.d., standard deviation. n, number.
Footnotes. 1, adjusted for baseline value and school of attendance. **2,** median calcium intake 466 mg/day. **3,** deseasonalised values.

Confidential: For Review Only

Table S4. Spirometric outcomes at 3-year follow-up by allocation, sub-study participants: overall and by sub-group

		Vitamin D: mean value (s.d.) [n]	Placebo: mean value (s.d.) [n]	Adjusted mean difference (95% CI)[1]	P	P for interaction
**% predicted FEV1**						
Overall		81.46 (13.37) [200]	82.48 (14.54) [187]	-1.68 (-4.23 to 0.88)	0.20	
By sex	Boys	80.33 (11.53) [95]	83.15 (12.94) [89]	-2.95 (-6.19 to 0.29)	0.07	0.16
	Girls	82.48 (14.81) [105]	81.87 (15.90) [98]	-0.50 (-4.30 to 3.31)	0.80	
By calcium intake[2]	<median	82.97 (12.53) [92]	83.31 (15.19) [97]	-1.40 (-4.98 to 2.18)	0.44	0.95
	≥median	80.58 (13.85) [102]	81.79 (13.90) [87]	-1.44 (-5.21 to 2.33)	0.45	
By baseline 25(OH)D concentration[3]	<75 nmol/L	82.26 (11.99) [103]	82.98 (14.21) [95]	-0.34 (-3.72 to 3.05)	0.84	0.10
	≥75 nmol/L	80.10 (12.14) [59]	84.34 (14.23) [54]	-4.80 (-9.12 to -0.49)	0.03	
**% predicted FVC**						
Overall		86.74 (13.53) [200]	87.10 (13.51) [187]	0.23 (-2.30 to 2.76)	0.86	
By sex	Boys	87.77 (12.16) [95]	90.36 (11.64) [89]	-2.17 (-5.37 to 1.03)	0.18	0.049
	Girls	85.81 (14.65) [105]	84.13 (14.43) [98]	2.11 (-1.73 to 5.95)	0.28	
By calcium intake[2]	<median	87.51 (12.47) [92]	87.15 (13.86) [97]	0.14 (-3.40 to 3.68)	0.94	0.97
	≥median	86.06 (14.05) [102]	87.19 (13.33) [87]	0.62 (-3.08 to 4.33)	0.74	
By baseline 25(OH)D concentration[3]	<75 nmol/L	86.67 (12.90) [103]	87.19 (13.84) [95]	-0.22 (-3.73 to 3.30)	0.90	0.92
	≥75 nmol/L	87.08 (12.79) [59]	86.85 (11.53) [54]	0.61 (-3.42 to 4.65)	0.77	
**% predicted FEV1/FVC**						
Overall		96.98 (14.02) [200]	96.99 (10.60) [187]	-0.84 (-3.30 to 1.62)	0.50	
By sex	Boys	97.31 (15.28) [95]	96.86 (10.03) [89]	0.02 (-3.64 to 3.69)	0.99	0.67
	Girls	96.69 (12.83) [105]	97.12 (11.14) [98]	-1.32 (-4.58 to 1.94)	0.43	
By calcium intake[2]	<median	97.64 (11.71) [92]	97.96 (9.54) [97]	-0.47 (-3.46 to 2.52)	0.76	0.95
	≥median	96.80 (15.07) [102]	96.12 (11.70) [87]	-1.42 (-5.26 to 2.43)	0.47	
By baseline 25(OH)D concentration[3]	<75 nmol/L	97.39 (10.36) [103]	97.39 (10.73) [95]	-0.38 (-3.26 to 2.50)	0.80	0.16
	≥75 nmol/L	95.63 (13.96) [59]	99.44 (9.48) [54]	-4.47 (-8.82 to -0.12)	0.04	

Abbreviations: 25(OH)D, 25-hydroxyvitamin D. FEV1, forced expiratory volume in 1 second. FVC, forced vital capacity. CI, confidence interval. S.d., standard deviation. n, number.

Footnotes. 1, adjusted for baseline value and school of attendance. **2,** median calcium intake 466 mg/day. **3,** deseasonalised values.

Confidential: For Review Only

BMJ Paediatrics Open

Influence of vitamin D supplementation on growth, body composition, pubertal development and spirometry in South African schoolchildren: a randomised controlled trial (ViDiKids)

Journal:	*BMJ Paediatrics Open*
Manuscript ID	bmjpo-2024-002495.R1
Article Type:	Original research
Date Submitted by the Author:	17-Feb-2024
Complete List of Authors:	Middelkoop, Keren; University of Cape Town; University of Cape Town, Department of Medicine<br>Micklesfield, Lisa; Faculty of Health Sciences University of Cape Town, Department of Human Biology; Faculty of Health Sciences University of the Witwatersrand, Department of Paediatrics<br>Stewart, Justine; University of Cape Town; University of Cape Town, Department of Medicine<br>Walker, Neil; Queen Mary University of London<br>Jolliffe, David A; Queen Mary University of London<br>Mendham, Amy E; Faculty of Health Sciences University of Cape Town, Department of Human Biology; Faculty of Health Sciences University of the Witwatersrand, Department of Paediatrics<br>Coussens, Anna K; Faculty of Health Sciences University of Cape Town; Walter and Eliza Hall Institute of Medical Research<br>Nuttall, James; University of Cape Town, the Department of Paediatrics and Child Health<br>Momand, Waheedullah; Queen Mary University of London<br>Wilkinson, Robert J; Faculty of Health Sciences University of Cape Town; Francis Crick Institute<br>Bekker, Linda-Gail; University of Cape Town; University of Cape Town, Department of Medicine<br>Tang, Jonathan ; University of East Anglia Faculty of Medicine and Health Sciences, Department of Medicine Norwich Medical School, Faculty of Medicine and Health Sciences, University of East Anglia, Norwich Research Park,<br>Fraser, William; Norwich Medical School, University of East Anglia, Faculty of Medicine and Health Sciences<br>cooper, cyrus; University of Southampton<br>Harvey, Nick; Mr.C Epidemiology Resource Centre<br>Martineau, Adrian; Queen Mary University of London
Keywords:	Adolescent Health, Growth

SCHOLARONE™
Manuscripts

I, the Submitting Author has the right to grant and does grant on behalf of all authors of the Work (as defined in the below author licence), an exclusive licence and/or a non-exclusive licence for contributions from authors who are: i) UK Crown employees; ii) where BMJ has agreed a CC-BY licence shall apply, and/or iii) in accordance with the terms applicable for US Federal Government officers or employees acting as part of their official duties; on a worldwide, perpetual, irrevocable, royalty-free basis to BMJ Publishing Group Ltd ("BMJ") its licensees and where the relevant Journal is co-owned by BMJ to the co-owners of the Journal, to publish the Work in this journal and any other BMJ products and to exploit all rights, as set out in our licence.

The Submitting Author accepts and understands that any supply made under these terms is made by BMJ to the Submitting Author unless you are acting as an employee on behalf of your employer or a postgraduate student of an affiliated institution which is paying any applicable article publishing charge ("APC") for Open Access articles. Where the Submitting Author wishes to make the Work available on an Open Access basis (and intends to pay the relevant APC), the terms of reuse of such Open Access shall be governed by a Creative Commons licence – details of these licences and which Creative Commons licence will apply to this Work are set out in our licence referred to above.

Other than as permitted in any relevant BMJ Author's Self Archiving Policies, I confirm this Work has not been accepted for publication elsewhere, is not being considered for publication elsewhere and does not duplicate material already published. I confirm all authors consent to publication of this Work and authorise the granting of this licence.

Confidential: For Review Only

Influence of vitamin D supplementation on growth, body composition, pubertal development and spirometry in South African schoolchildren: a randomised controlled trial (ViDiKids)

Keren Middelkoop[1,2*]
Lisa K Micklesfield[3,4*]
Justine Stewart[1,2]
Neil Walker[5]
David A Jolliffe[6]
Amy E Mendham[3,4]
Anna K Coussens[7,8]
James Nuttall[9]
Jonathan C Y Tang[10,11]
William D Fraser[10,11]
Waheedullah Momand[6]
Cyrus Cooper[12,13]
Nick Harvey[12,13]
Robert J Wilkinson[7,14,15]
Linda-Gail Bekker[1,2]
Adrian R Martineau[6†]

1, Desmond Tutu HIV Centre, Institute of Infectious Disease & Molecular Medicine, University of Cape Town, Observatory, South Africa
2, Department of Medicine, University of Cape Town, Observatory, South Africa
3, Health through Physical Activity, Lifestyle and Sport Research Centre (HPALS), Division of Physiological Sciences, Department of Human Biology, Faculty of Health Sciences, University of Cape Town, Cape Town, South Africa
4, SAMRC/Wits Developmental Pathways for Health Research Unit, Department of Paediatrics, Faculty of Health Sciences, University of the Witwatersrand, Johannesburg, South Africa
5, Wolfson Institute of Population Health, Barts and The London School of Medicine and Dentistry, Queen Mary University of London, London, UK
6, Blizard Institute, Barts and The London School of Medicine and Dentistry, Queen Mary University of London, London, UK

Confidential: For Review Only

7, Centre for Infectious Diseases Research in Africa and Institute of Infectious Disease and Molecular Medicine, Faculty of Health Sciences, University of Cape Town, Observatory 7925, South Africa
8, Infectious Diseases and Immune Defence Division, Walter and Eliza Hall Institute of Medical Research, Parkville, Australia
9, Paediatric Infectious Diseases Unit, Red Cross War Memorial Children's Hospital and the Department of Paediatrics and Child Health, University of Cape Town, Cape Town, South Africa
10, Norwich Medical School, University of East Anglia, Norwich Research Park, Norwich, UK
11, Departments of Laboratory Medicine, Clinical Biochemistry and Departments of Diabetes and Endocrinology, Norfolk and Norwich University Hospital NHS Foundation Trust, Norwich, UK
12, MRC Lifecourse Epidemiology Centre, University of Southampton, Southampton UK
13, NIHR Southampton Biomedical Research Centre, University of Southampton, Southampton UK and University Hospital Southampton NHS Foundation Trust, Southampton, UK
14, The Francis Crick Institute, London, NW1 1AT, UK
15, Department of Infectious Diseases, Imperial College London, London, W12 0NN, UK

* These authors contributed equally
†To whom correspondence should be addressed at the Blizard Institute, Barts and The London School of Medicine and Dentistry, Queen Mary University of London, 4 Newark St, London E1 2AT, UK; Tel: +44 207 882 7242; Email: a.martineau@qmul.ac.uk

Funding

This research was funded by the UK Medical Research Council (refs MR/R023050/1 and MR/M026639/1, both awarded to ARM).

Confidential: For Review Only

ABSTRACT

Objective: To determine whether weekly oral vitamin D supplementation influences growth, body composition, pubertal development or spirometric outcomes in South African schoolchildren.

Design: Phase 3 double-blind randomised placebo-controlled trial (clinicaltrials.gov registration no. NCT02880982).

Setting: Socio-economically disadvantaged peri-urban district of Cape Town, South Africa

Participants: 1682 children of Black African ancestry attending government primary schools and aged 6-11 years at baseline.

Interventions: Oral vitamin D_3 (10,000 IU/week) vs. placebo for 3 years

Main outcome measures: height-for-age and body mass index-for-age, measured in all participants); Tanner scores for pubertal development, spirometric lung volumes and body composition, measured in a subset of 450 children who additionally took part in a nested sub-study.

Results: Mean serum 25-hydroxyvitamin D_3 concentration at 3-year follow-up was higher among children randomised to receive vitamin D vs. placebo (104.3 vs. 64.7 nmol/L, respectively; mean difference [MD] 39.7 nmol/L, 95% CI 37.6 to 41.9 nmol/L). No statistically significant differences in height-for-age z-score (adjusted MD [aMD] -0.08, 95% CI -0.19 to 0.03) or body mass index-for-age z-score (aMD -0.04, 95% CI -0.16 to 0.07) were seen between vitamin D vs. placebo groups at follow-up. Among sub-study participants, allocation to vitamin D vs. placebo did not influence pubertal development scores, % predicted forced expiratory volume in 1 second (FEV1), % predicted forced vital capacity (FVC), % predicted FEV1/FVC, fat mass or fat-free mass.

Conclusions: Weekly oral administration of 10,000 IU vitamin D_3 boosted vitamin D status but did not influence growth, body composition, pubertal development or spirometric outcomes in South African schoolchildren.

Confidential: For Review Only

KEY MESSAGES

What is already known on this topic?

- Observational studies have reported independent associations between vitamin D deficiency in childhood and slower linear growth, reduced lean mass, obesity and precocious puberty.
- A phase 2 clinical trial conducted in Mongolia reported that a 6-month course of vitamin D supplementation increased height gain in 113 vitamin D deficient schoolchildren aged 12-15 years; however, these results were not confirmed by a recent phase 3 trial conducted in the same setting.
- RCTs to determine effects of vitamin D supplementation on growth and development in schoolchildren have not been conducted in other settings.

What this study adds

- This placebo-controlled phase 3 clinical trial, conducted in 1682 Black African schoolchildren in Cape Town, South Africa, showed that a 3-year course of weekly vitamin D supplementation was effective in elevating circulating 25-hydroxyvitamin D concentrations.
- However, this was not associated with any effect on linear growth, body composition, pubertal development or spirometric lung volumes.

How this study might affect research, practice or policy

- Our findings do not support use of vitamin D supplementation as an intervention to influence child growth, body composition, pubertal development or spirometric lung volumes.

Confidential: For Review Only

INTRODUCTION

Middle childhood and early adolescence represent key periods for growth and development that has an important influence on stature and health outcomes in later adolescence and adulthood.[1] Stunting in childhood has long been recognised to associate with multiple adverse long-term health outcomes,[2] and is particularly common in lower-income countries.[3] These settings have also witnessed an emerging epidemic of childhood obesity,[4] which has in turn been associated with accelerated pubertal development.[5] Interventions to alleviate these public health challenges are urgently needed.

Vitamin D is a fat-soluble micronutrient with pleotropic effects on human health.[6] Observational studies have reported that vitamin D deficiency, as evidenced by low circulating concentrations of its major metabolite 25-hydroxyvitamin D (25[OH]D), associates with slower linear growth,[7] reduced lean mass,[8] childhood obesity[9-11] and precocious puberty,[12, 13] potentially reflecting the ability of vitamin D to stimulate production of insulin-like growth factor 1[14] and regulate adipogenesis.[15] A phase 2 randomised controlled trial (RCT) conducted in Mongolia reported that a 6-month course of vitamin D supplementation increased height gain in 113 vitamin D deficient schoolchildren aged 12-15 years,[16] but these results were not confirmed by a recent phase 3 RCT conducted in the same setting.[17] RCTs to determine effects of vitamin D supplementation on growth and development in schoolchildren have not been conducted in other settings representing children at different risks of malnutrition, stunting and obesity. An opportunity to conduct such an investigation recently arose as part of the ViDiKids trial, a multicentre phase 3 RCT which investigated the effects of weekly oral administration of 10,000 IU vitamin D_3 for 3 years on the primary outcome of tuberculosis infection in a cohort of 1682 schoolchildren aged 6-11 years living in a socio-economically disadvantaged peri-urban district of Cape Town, South Africa.[18] Height-for-age z-scores and body mass index-for age z-scores were assessed in all participants (n=1682), and Tanner scores for pubertal development, spirometric lung volumes and body composition were assessed in a subset of 450 children who also took part in a nested sub-study.

Confidential: For Review Only

METHODS

TRIAL DESIGN, SETTING, APPROVALS AND REGISTRATION

We conducted a multicentre phase 3 double-blind individually randomised placebo-controlled trial of weekly oral vitamin D supplementation in 23 government schools in Cape Town, South Africa, as previously described.[18, 19] The primary outcome was acquisition of tuberculosis infection, as evidenced by conversion of a QuantiFERON-TB Gold Plus (QFT-Plus) assay result from negative at baseline to positive at 3-year follow-up. The current manuscript reports effects of the intervention on pre-specified secondary outcomes relating to growth in all study participants, and body composition, pubertal development and spirometry in a subset of participants who additionally took part in a nested sub-study. The trial was sponsored by Queen Mary University of London and registered on the South African National Clinical Trials Register (DOH-27-0916-5527) and ClinicalTrials.gov (ref NCT02880982).

RESEARCH ETHICS APPROVAL

The trial was approved by the University of Cape Town Faculty of Health Sciences Human Research Ethics Committee (Ref: 796/2015) and the London School of Hygiene and Tropical Medicine Observational/Interventions Research Ethics Committee (Ref: 7450-2)

PARTICIPANTS

Inclusion criteria for the main trial were enrolment in Grades 1-4 at a participating school; age 6 to 11 years at screening; and written informed assent / consent to participate in the main trial provided by children and their parent / legal guardian, respectively. Exclusion criteria for the main trial were a history of previous tuberculosis infection, active TB disease or any chronic illness other than asthma (including known or suspected HIV infection) prior to enrolment; use of any regular medication other than asthma medication; use of vitamin D supplements at a dose of more than 400 IU/day in the month before enrolment; plans to move away from study area within 3 years of enrolment; inability to swallow a placebo soft gel capsule with ease; and clinical evidence of rickets or a positive QFT-Plus assay result at screening. An

Confidential: For Review Only

additional inclusion criterion for the sub-study was enrolment in Grade 4 at a participating school (i.e. only children in grade 4 were eligible for the sub-study).

ENROLMENT

Full details of enrolment procedures are described in Supplementary Methods (Supplementary Material). Eligible participants underwent measurement of weight using a digital floor scale (Charder Medical, Taichung City, Taiwan), height using a portable stadiometer (HM200P, Charder Medical) and waist circumference using a measuring tape. Sub-study participants also underwent spirometry according to ERS/ATS standards[20] using a portable spirometer (Carefusion, San Diego, CA) and measurement of body composition by dual energy x-ray absorptiometry (DXA) as described below.

RANDOMISATION AND BLINDING

Full details of randomisation and blinding procedures have been described previously,[18, 19] and are presented in Supplementary Methods (Supplementary Material). Briefly, eligible and assenting children whose parents consented to their participation in the trial were individually randomised to receive a weekly capsule containing vitamin D_3 or placebo for three years, with a one-to-one allocation ratio and randomisation stratified by school of attendance. Treatment allocation was concealed from participants, care providers and all trial staff (including senior investigators and those assessing outcomes) until completion of the trial to maintain the double-blind.

INTERVENTION

Study medication comprised a 3-year course of weekly soft gel capsules manufactured by the Tishcon Corporation (Westbury, NY, USA), containing either 0.25 mg (10,000 international units) cholecalciferol (vitamin D_3) in olive oil (intervention arm) or olive oil without any vitamin D_3 content (placebo arm). A weekly dose of 10,000 IU (equivalent to 1,429 IU/day) was selected in preference to a daily dose of 600 IU (the RDA for this age group) because we were concerned that the latter dose would be inadequate to maintain serum 25(OH)D concentrations >50 nmol/L,[21] and we felt that adherence

Confidential: For Review Only

to a directly-observed weekly dose would be superior to daily self-administration.[22] Weekly supplementation has also been shown to be effective in elevating 25(OH)D concentrations into the physiological range in children by other investigators.[23] Active and placebo capsules had identical appearance and taste. Capsules were taken under direct observation of study staff during school term time. Further details of administration of study medication are provided in Supplementary Methods (Supplementary Material).

FOLLOW-UP ASSESSMENTS

At 1-year, 2-year and 3-year follow-up, height, weight and waist circumference were measured as at baseline. At 3-year follow-up, sub-study participants were additionally invited to undergo repeat spirometry and DXA scanning, and to complete a Tanner self-assessment questionnaire for pubertal development.[24]

OUTCOMES

The primary outcome for the main trial, reported elsewhere,[18] was the QFT-Plus result at the end of the study. The following secondary outcomes were assessed for all participants: height-for-age, BMI-for-age, waist circumference-for-age and waist-to-height ratio (all participants). Additional outcomes assessed in sub-study participants only were: whole body fat mass, fat-free soft tissue mass, % predicted forced vital capacity (FVC), % predicted forced expiratory volume in one second (FEV1), % predicted FEV1/FVC, mean Tanner scores for pubic hair (males and females), external genitalia (males only) or breast development (females only), the proportion of participants reaching menarche by the end of the trial (females only) and mean age at menarche (females who reached menarche by the end of the trial only).

DXA

Body composition (whole body fat mass and fat-free soft tissue mass) was assessed using a Hologic Discovery-W® dual energy x-ray absorptiometry (DXA) scanner at the Sports Science Institute of South Africa, University of Cape Town. All scans were performed by a trained radiographer on one scanner (Hologic, Bedford, MA, USA) using standard procedures, and analysed using Apex software (Version 13.4.1). Quality assurance checks were carried out prior to scanning and generated

Confidential: For Review Only

coefficients of variation <0.5%. DXA outcomes relating to effects of the trial intervention on bone mineral content have been reported elsewhere.[19]

LABORATORY ASSESSMENTS

Serum concentrations of 25(OH)D_3 were measured using liquid chromatography tandem mass spectrometry (LC-MS/MS) as previously described.[25] Further details are presented in Supplementary Methods (Supplementary Material).

SAMPLE SIZE

Sample size for the main trial was calculated as the number needed to detect a 25% reduction in the proportion of children with a positive QFT-Plus assay result at 3-year follow-up (primary outcome) with 80% power and 5% type 1 error, assuming a 3.5% annual risk of QFT-Plus conversion, 20% loss to follow- up, and a 5% risk of an indeterminate QFT-Plus assay result at the end of the study.[18] Sample size for the sub-study was calculated as the number needed detect an inter-arm difference of 0.35 standard deviations in mean bone mineral content at the whole body less head and lumbar spine with 88% power and 5% type 1 error, assuming 29% loss to follow-up at 3 years.[19]

STATISTICAL ANALYSES

Full details of statistical analyses are provided in Supplementary Methods (Supplementary Material). Briefly, effects of treatment on age- and sex-adjusted z-scores for anthropometric outcomes were estimated by fitting allocation to vitamin D vs. placebo as the sole fixed effect in a mixed effects linear regression model with a random effect for repeated assessments of each individual participant and a random effect of school of attendance. Effects of treatment on DXA and spirometric outcomes were analysed using multi-level mixed models with adjustment for baseline values and a random effect for school. Tanner metrics were analysed in a similar fashion, but restricted to the applicable sex and without baseline adjustment. Pre-specified sub-group analyses were conducted to determine whether the effect of vitamin D supplementation was modified by sex (male vs. female), baseline deseasonalised 25(OH)D_3 concentration (<75 vs. ≥75 nmol/L)[26] and calcium intake (< vs. ≥ median value of 466 mg/day).[19] These were performed by repeating efficacy analyses with the inclusion of an interaction term between allocation (to vitamin D vs. placebo) and

Confidential: For Review Only

each posited effect-modifier with presentation of the P-value associated with this interaction term. An Independent Data Monitoring Committee reviewed accumulating serious adverse event data at 6-monthly intervals, and recommended continuation of the trial at each review. No interim efficacy analysis was performed.

PATIENT AND PUBLIC INVOLVEMENT

The Desmond Tutu HIV Centre's Community Advisory Group were consulted on the design and conduct of the ViDiKids trial.

Confidential: For Review Only

RESULTS

PARTICIPANTS

A total of 2852 children were screened for eligibility from March 2017 to March 2019, of whom 2271 underwent QFT testing: 1682 (74.1%) QFT-negative children were randomly assigned to receive vitamin D_3 (829 participants) or placebo (853 participants) as previously described.[18] 450/1682 (26.8%) participants in the main trial also participated in the sub-study, of whom 228 vs. 222 participants were allocated to the vitamin D vs. placebo arms, respectively (Fig. 1). Table 1 presents baseline characteristics of children in the main trial and in the sub-study, overall and by study arm. Mean age was higher among participants in the sub-study vs. all those in the main trial (10.1 vs. 8.9 years, respectively), reflecting the fact that participation in the sub-study was restricted to children enrolled in Grade 4. Prevalence of obesity and stunting were lower in the sub-study vs. the main trial (3.8% vs 5.5%, and 6.4% vs. 11.1%, respectively. Baseline characteristics were otherwise well balanced for all participants in the main trial vs. those who additionally participated in the sub-study: 52.4% vs. 52.0% were female and mean serum 25(OH)D_3 concentrations were 71.2 nmol/L vs. 70.0 nmol/L. Within the main trial and the sub-study, baseline characteristics of those randomised to vitamin D vs. placebo were also well balanced. The median duration of follow-up was 3.16 years (interquartile range, 2.83 to 3.38 years) and was not different between the two study arms. For the main trial, mean serum 25(OH)D_3 concentrations at 3-year follow-up were higher among children randomised to receive vitamin D vs. placebo (104.3 vs. 64.7 nmol/L, respectively; mean difference 39.7 nmol/L, 95% CI for difference 37.6 to 41.9 nmol/L).

GROWTH OUTCOMES

Among participants in the main trial, allocation to vitamin D vs. placebo did not significantly influence mean height-for-age z-scores at annual follow-up, either overall or within sub-groups defined by sex, calcium intake or baseline 25(OH)D concentration (Table 2: P values for interaction >0.05). Similarly, no statistically significant effect of the intervention was seen on mean BMI-for-age z-scores (Table 3), waist circumference-for-age z-scores (Table S1) or waist-to-height ratio z-scores (Table

Confidential: For Review Only

S2), either overall or by sub-group (P values for interaction >0.05). Among sub-study participants, allocation to vitamin D vs. placebo did not significantly influence fat mass or fat-free soft tissue mass (Table S3) or % predicted FEV1 or FEV1/FVC (Table S4), either overall or by sub-group. For the outcome of % predicted FVC, allocation to vitamin D vs. placebo did not have a statistically significant effect overall, or within sub-groups defined by calcium intake or baseline 25(OH)D concentration; however, the P value for interaction associated with the sub-group analysis by sex (P=0.049) raised the possibility that this factor might modify the effect of vitamin D on % predicted FVC (Table S4).

DEVELOPMENTAL OUTCOMES

Among sub-study participants, no statistically significant inter-arm differences were seen in mean Tanner scores for pubic hair (males and females), external genitalia (males only), proportion menstruating (females only), mean age at menarche (females only), or breast development (females only), either overall or within sub-groups defined by calcium intake or baseline 25(OH)D concentration (Table 4).

ADVERSE EVENTS

Incidence of adverse events by trial arm has been reported elsewhere.[18] No serious events arising in the trial were adjudged related to administration of vitamin D or placebo.

Confidential: For Review Only

DISCUSSION

We report findings from the first randomised controlled trial to investigate effects of vitamin D supplementation on growth and developmental indices in schoolchildren of Black African ancestry. Administration of oral vitamin D supplementation at a dose of 10,000 IU per week for 3 years was effective in boosting vitamin D status, but it did not have statistically significant effects on linear growth, body mass index, body composition, spirometric lung volumes or self-assessed pubertal development, either overall or in sub-groups defined by sex, calcium intake or baseline vitamin D status.

Null findings from the current study contrast with those from observational studies reporting independent associations between vitamin D deficiency and reduced lean mass,[8] slower linear growth,[7] childhood obesity[9-11] and precocious puberty.[12] However, they are consistent with those of the only other phase 3 RCT to investigate effects of vitamin D on growth and development in school-age children that we are aware of, which reported no effect of weekly vitamin D supplementation on growth or development among Mongolian schoolchildren aged 6-13 years at baseline.[17] Contrasting findings from observational studies vs. clinical trials may reflect the fact that the former are more susceptible to effects of confounding or reverse causality. Although we observed limited evidence (P for interaction 0.049) to support the hypothesis that effects of vitamin D on FVC might be modified by sex, this finding may have arisen because of type 1 error, given the multiplicity of analyses conducted.

Our study has several strengths. The intervention was sustained (3 years), allowing ample time for any effects of vitamin D supplementation on outcomes of interest to manifest. Moreover, the intervention was effective in elevating serum 25(OH)D concentrations, reflecting adequacy of the dosing regimen employed as well as good adherence resulting from directly observed administration of weekly supplements in schools during termtime. Our large sample size and low rates of loss to follow-up maximised power to detect modest effects of the intervention, particularly for outcomes that were assessed in the main trial population. Furthermore, we assessed a broad range of anthropometric and developmental outcomes, that included use of DXA, the gold standard investigation for assessment of body composition.

Confidential: For Review Only

Our study also has some limitations. Baseline prevalence of vitamin D deficiency was low, perhaps reflecting plentiful exposure to sunshine in the study setting.[25] Dietary factors are less likely to contribute to this phenomenon, as frequency of intake of oily fish and other foods containing vitamin D did not associate with baseline vitamin D status in the study population.[25] Our results cannot therefore be generalised to settings where vitamin D deficiency is common. However, we highlight that results from our trial in Mongolia (where baseline prevalence of vitamin D deficiency was much higher than we observed in the current study) were also null for outcomes of linear growth and body mass index.[17] Accordingly, null results from the current trial cannot be explained simply by the relatively high baseline vitamin D status of participants in Cape Town. A second potential limitation relates to the fact that pubertal status was assessed by participants themselves, rather than by health professionals, whose judgement may be more objective. However, the double-blind placebo-controlled trial design of our study will have distributed any resultant imprecision equally between study arms, ensuring that bias was not introduced. Moreover, pubertal self-assessment has previously been done by adolescent participants in another South African cohort study, and shown to be reliable.[27]

In conclusion, we report that oral vitamin D supplementation at a dose of 10,000 IU/week for 3 years was effective in elevating serum 25(OH)D concentrations in schoolchildren of Black African ancestry living in Cape Town, South Africa. However, this was not associated with any effects on linear growth, body habitus, spirometric outcomes or pubertal development. Taken together with null results from a trial of weekly vitamin D supplementation with similar outcomes conducted in Mongolia,[17] our findings do not support the use of vitamin D supplements to influence child growth or development in populations where rickets has been excluded. Further research could be conducted to re-evaluate growth and developmental outcomes in the future, to determine whether any adverse effects might be associated with withdrawing vitamin D supplementation from individuals randomised to the intervention arm of this study, who may have become habituated to it during their participation in the trial.

ACKNOWLEDGEMENTS

Confidential: For Review Only

We thank all the children who participated in the trial, and their parents / guardians; members of the Independent Data Monitoring Committee (Prof Guy Thwaites, Oxford University Clinical Research Unit, Ho Chi Minh City, Vietnam [Chair]; Prof John Pettifor, University of the Witwatersrand, Johannesburg, South Africa; and Prof Sarah Walker, MRC Clinical Trials Unit, London, UK); and members of the Trial Steering Committee (Prof Beate Kampmann, London School of Hygiene and Tropical Medicine, London, UK [Chair]; Prof Ashraf Coovadia, University of the Witwatersrand, Johannesburg, South Africa; Dr Karen Jennings, City Health, Cape Town, South Africa; Dr Robin Dyers, Department of Health, Western Cape Government, Cape Town, South Africa; and Dr Guy de Bruyn, Sanofi Pasteur, Swiftwater PA USA). For the purposes of open access the author has applied a CC-BY public copyright to any author-accepted manuscript arising from this submission.

CONTRIBUTORS

ARM conceived the study. KM, LKM, AKC, JN, CC, NCH, RLH, RJW, LGB and ARM contributed to study design and protocol development. KM led on trial implementation, with support from JS, CD, DAJ, JN, LGB and ARM. LKM and AEM oversaw performance of DXA scans. JCYT and WDF performed and supervised the conduct of biochemical assays. NW, RLH and ARM drafted the statistical analysis plan. DAJ, KM, JS, NW, WM and CD managed data. NW accessed, verified and analysed the data underlying the study. ARM and DAJ wrote the first draft of the trial report. All authors made substantive comments thereon and approved the final version for submission.

COMPETING INTERESTS

ARM declares receipt of funding in the last 36 months to support vitamin D research from the following companies who manufacture or sell vitamin D supplements: Pharma Nord Ltd, DSM Nutritional Products Ltd, Thornton & Ross Ltd and Hyphens Pharma Ltd. ARM also declares receipt of vitamin D capsules for clinical trial use from Pharma Nord Ltd, Synergy Biologics Ltd and Cytoplan Ltd; support for attending meetings from Pharma Nord Ltd and Abiogen Pharma Ltd; receipt of consultancy fees from DSM Nutritional Products Ltd and Qiagen Ltd; receipt of a speaker fee from the Linus Pauling Institute; participation on Data and Safety Monitoring Boards for the VITALITY

Confidential: For Review Only

trial (Vitamin D for Adolescents with HIV to reduce musculoskeletal morbidity and immunopathology, Pan African Clinical Trials Registry ref PACTR20200989766029) and the Trial of Vitamin D and Zinc Supplementation for Improving Treatment Outcomes Among COVID-19 Patients in India (ClinicalTrials.gov ref NCT04641195); and unpaid work as a Programme Committee member for the Vitamin D Workshop. All other authors declare that they have no competing interests.

DATA AVAILABILITY STATEMENT

Anonymised data may be requested from the corresponding author to be shared subject to terms of research ethics committee approval.

Confidential: For Review Only

Table 1: Participants' baseline characteristics by allocation: main study and sub-study

		Main study (n=1682)			Sub-study (n=450 subset)		
		**Overall (n=1,682)**	**Vitamin D arm (n=829)**	**Placebo arm (n=853)**	**Overall (n=450)**	**Vitamin D arm (n=228)**	**Placebo arm (n=222)**
Mean age, years (s.d.)		8.9 (1.4)	8.9 (1.4)	8.8 (1.3)	10.1 (0.7)	10.2 (0.7)	10.0 (0.6)
Female sex, n (%)		880 (52.4)	437 (52.8)	443 (51.9)	234 (52.0)	116 (50.9)	118 (53.2)
Ethnic origin[1]	Xhosa, n (%)	1615 (97.9)	788 (97.3)	827 (98.5)	424 (96.4)	214 (96.0)	210 (96.8)
	Other, n (%)	35 (2.1)	22 (2.7)	13 (1.5)	16 (3.6)	9 (4.0)	7 (3.2)
Type of residence	Brick, n (%)	867 (51.5)	423 (51.0)	444 (52.1)	230 (51.1)	121 (53.1)	109 (49.1)
	Informal, n(%)	815 (48.5)	406 (49.0)	409 (47.9)	220 (48.9)	107 (46.9)	113 (50.9)
Parental education[1,2]	Primary school, n (%)	60 (3.6)	34 (4.1)	26 (3.1)	24 (5.3)	16 (7.0)	8 (3.6)
	Secondary school or higher, n (%)	1618 (96.4)	792 (95.9)	826 (96.9)	426 (94.7)	212 (93.0)	214 (96.4)
Mean monthly household income, 1,000 ZAR (s.d.)		1.88 (2.20)	1.81 (2.15)	1.95 (2.25)	1.57 (2.31)	1.52 (2.69)	1.63 (1.86)
Mean height-for-age z-score (s.d.)[1]		-0.55 (1.20)	-0.56 (1.26)	-0.54 (1.15)	-0.43 (0.96)	-0.43 (0.98)	-0.44 (0.95)
Height-for-age z-score category	<-2.00 (stunted), n (%)	186 (11.1)	99 (11.9)	87 (10.2)	29 (6.4)	16 (7.0)	13 (5.9)
	≥ -2.00, n (%)	1496 (88.9)	730 (88.1)	766 (89.8)	421 (93.6)	212 (93.0)	209 (94.1)
Mean BMI-for-age z-score (s.d.)[1]		0.33 (1.03)	0.31 (1.03)	0.34 (1.03)	0.20 (0.97)	0.19 (0.92)	0.21 (1.02)
BMI-for-age z-score category	<-2.00 (thin), n (%)	26 (1.5)	15 (1.8)	11 (1.3)	7 (1.6)	4 (1.8)	3 (1.4)
	> -2.00 & ≤1.00, n (%)	1223 (72.7)	602 (72.6)	621 (72.8)	354 (78.7)	178 (78.1)	176 (79.3)
	>1.00 & ≤2.00 (overweight), n (%)	340 (20.2)	165 (19.9)	175 (20.5)	72 (16.0)	42 (18.4)	30 (13.5)
	>2.00 (obese), n (%)	93 (5.5)	47 (5.7)	46 (5.4)	17 (3.8)	4 (1.8)	13 (5.9)
Mean waist circumference-for-age z-score (s.d.)[1]		-0.13 (0.90)	-0.11 (0.90)	-0.15 (0.91)	-0.20 (0.98)	-0.20 (0.98)	-0.20 (0.98)
Mean waist-to-height ratio z-score (s.d.)[1]		0.03 (0.85)	0.05 (0.83)	0.00 (0.86)	-0.05 (0.86)	-0.05 (0.85)	-0.04 (0.88)
Mean whole body fat mass, kg (s.d.)		--	--	--	3.71 (2.26)	3.66 (2.13)	3.77 (2.40)
Mean whole body fat-free soft tissue mass, kg (s.d.)		--	--	--	9.27 (1.95)	9.39 (2.02)	9.15 (1.86)
% predicted FEV1 (s.d.)		--	--	--	83.2 (15.7)	83.8 (15.2)	82.7 (16.2)
% predicted FVC (s.d.)		--	--	--	94.8 (18.7)	94.6 (18.2)	95.0 (19.3)
% predicted FEV1/FVC (s.d.)		--	--	--	91.4 (16.9)	92.2 (16.3)	90.5 (17.5)
Calcium intake[1]	<median, n (%)[3]	817 (50.0)	383 (47.6)	434 (52.3)	248 (56.2)	119 (53.6)	129 (58.9)
	≥median, n (%)[3]	817 (50.0)	421 (52.4)	396 (47.7)	193 (43.8)	103 (46.4)	90 (41.1)
Mean serum 25(OH)D_3 concentration, nmol/L (s.d.)[1,4]		71.2 (14.8)	71.2 (14.5)	71.1 (15.0)	70.0 (13.5)	70.4 (12.1)	69.6 (14.9)
Serum 25(OH)D_3 concentration, category[1,4]	<25 nmol/L, n (%)	1 (0.1)	0 (0.0)	1 (0.1)	1 (0.3)	0 (0)	1 (0.6)
	≥ 25 nmol/L & < 50 nmol/L, n (%)	74 (5.4)	34 (5.1)	40 (5.8)	20 (5.5)	7 (3.7)	13 (7.4)
	≥ 50 nmol/L & < 75 nmol/L, n (%)	787 (57.7)	394 (58.8)	393 (56.6)	214 (59.0)	114 (61.0)	100 (56.8)
	≥75 nmol/L, n (%)	502 (36.8)	242 (36.1)	260 (37.5)	128 (35.3)	66 (35.3)	62 (35.2)

Confidential: For Review Only

Abbreviations: $25(OH)D_3$, 25-hydroxyvitamin D_3. ALP, alkaline phosphatase. BMI, body mass index. BMC, bone mineral content. CTX, C-terminal cross-linked telopeptide; P1NP, serum procollagen type I N-propeptide. S.d., standard deviation. ZAR, South African Rand.

Footnotes. 1, missing data (for bone sub-study: ethnicity, n=5 vitamin D arm, n=5 placebo arm; for calcium intake, n=6 Vitamin D arm, n=3 placebo arm; for serum $25(OH)D_3$ concentration, n=66 vitamin D arm, n=62 placebo arm; for serum adjusted calcium and total ALP concentration, n=41 vitamin D arm, n=45 placebo arm; for PTH: n=42 vitamin D arm, n=46 placebo arm; for CTX: n=41 vitamin D arm, n=46 placebo arm; for P1NP: n=43 vitamin D arm, n=46 placebo arm; for fracture study: ethnicity, n=19 vitamin D arm, n=13 placebo arm; parental education, n=3 vitamin D arm, n=1 placebo arm; BMI-for-age z-score, n=2 vitamin D arm, n=0 placebo arm; height-for-age z-score, n=2 vitamin D arm, n=0 placebo arm; for calcium intake, n=25 Vitamin D arm, n=23 placebo arm; for serum $25(OH)D_3$ concentration, n=159 vitamin D arm, n=159 placebo arm; **2,** highest level of education of at least one parent. **3,** median calcium intake 466 mg/day. **4,** deseasonalised values

Confidential: For Review Only

Table 2. Mean height-for-age z-scores at annual follow-up by allocation, main study participants: overall and by sub-group

		Follow-up time point	Vitamin D arm: mean value (s.d.) [n]	Placebo arm: mean value (s.d.) [n]	Adjusted mean difference (95% CI)[1]	P for timepoint[1]	Overall P[1]	P for interaction[1]
Overall		1 year	-0.22 (1.12) [664]	-0.15 (1.07) [661]	-0.06 (-0.17 to 0.06)	0.33	0.22	--
		2 years	-0.14 (1.05) [613]	-0.06 (1.01) [606]	-0.04 (-0.16 to 0.07)	0.46		
		3 years	-0.26 (1.04) [670]	-0.14 (1.05) [691]	-0.08 (-0.19 to 0.03)	0.17		
By sex	Boys	1 year	-0.31 (1.14) [304]	-0.27 (1.06) [306]	-0.02 (-0.17 to 0.14)	0.85	0.42	0.94
		2 years	-0.21 (1.11) [285]	-0.19 (0.95) [283]	0.04 (-0.13 to 0.20)	0.67		
		3 years	-0.34 (1.07) [310]	-0.28 (1.00) [326]	-0.03 (-0.19 to 0.13)	0.69		
	Girls	1 year	-0.15 (1.09) [360]	-0.04 (1.07) [355]	-0.10 (-0.25 to 0.06)	0.21	0.34	
		2 years	-0.08 (0.99) [328]	0.05 (1.06) [323]	-0.12 (-0.28 to 0.04)	0.14		
		3 years	-0.19 (1.02) [360]	-0.02 (1.08) [365]	-0.13 (-0.28 to 0.03)	0.11		
By calcium intake[2]	<median	1 year	-0.16 (1.05) [314]	-0.10 (1.02) [331]	-0.04 (-0.18 to 0.11)	0.64	0.02	0.07
		2 years	-0.17 (0.96) [288]	-0.05 (0.96) [310]	-0.04 (-0.19 to 0.11)	0.64		
		3 years	-0.28 (0.99) [312]	-0.15 (0.99) [362]	-0.08 (-0.23 to 0.07)	0.29		
	≥median	1 year	-0.25 (1.16) [327]	-0.18 (1.13) [314]	-0.05 (-0.21 to 0.12)	0.60	0.72	
		2 years	-0.07 (1.12) [307]	-0.06 (1.06) [279]	-0.03 (-0.20 to 0.14)	0.76		
		3 years	-0.20 (1.09) [337]	-0.13 (1.11) [311]	-0.06 (-0.22 to 0.11)	0.50		
By baseline 25(OH)D concentration[3]	<75 nmol/L	1 year	-0.23 (1.13) [349]	-0.03 (1.11) [332]	-0.17 (-0.33 to -0.01)	0.04	0.53	0.50
		2 years	-0.14 (1.11) [325]	0.05 (1.06) [323]	-0.12 (-0.28 to 0.04)	0.15		
		3 years	-0.25 (1.10) [340]	-0.06 (1.07) [359]	-0.15 (-0.31 to 0.01)	0.07		
	≥75 nmol/L	1 year	-0.22 (1.15) [187]	-0.25 (0.98) [201]	0.02 (-0.17 to 0.22)	0.82	0.15	
		2 years	-0.17 (1.00) [171]	-0.08 (0.96) [179]	-0.07 (-0.26 to 0.13)	0.50		
		3 years	-0.30 (1.00) [196]	-0.18 (0.99) [206]	-0.06 (-0.25 to 0.13)	0.53		

Abbreviations: 25(OH)D, 25-hydroxyvitamin D. CI, confidence interval. S.d., standard deviation. n, number.

Footnotes. 1, effect estimates and P values from mixed effects linear regression models including a fixed effect for allocation to vitamin D vs. placebo, a random effect for repeated assessments of each individual participant and a random effect of school of attendance. **2,** median calcium intake 466 mg/day. **3,** deseasonalised values.

Confidential: For Review Only

Table 3. Mean BMI-for-age z-scores at annual follow-up by allocation, main study participants: overall and by sub-group

			**Vitamin D arm: mean value (s.d.) [n]**	**Placebo arm: mean value (s.d.) [n]**	**Adjusted mean difference (95% CI)[1]**	**P for timepoint[1]**	**Overall P[1]**	**P for interaction[1]**
Overall		1 year	0.16 (1.03) [664]	0.17 (1.02) [661]	-0.02 (-0.14 to 0.09)	0.68	0.86	--
		2 years	0.15 (1.41) [613]	0.25 (1.21) [606]	-0.09 (-0.20 to 0.03)	0.14		
		3 years	0.17 (1.07) [669]	0.21 (1.17) [691]	-0.04 (-0.16 to 0.07)	0.45		
By sex	Boys	1 year	0.08 (0.99) [304]	-0.00 (0.99) [306]	0.09 (-0.08 to 0.27)	0.30	0.59	0.29
		2 years	0.00 (1.68) [285]	-0.04 (1.30) [283]	0.04 (-0.14 to 0.22)	0.64		
		3 years	-0.02 (1.04) [309]	-0.12 (1.20) [326]	0.09 (-0.09 to 0.26)	0.33		
	Girls	1 year	0.23 (1.06) [360]	0.32 (1.02) [355]	-0.14 (-0.28 to 0.00)	0.06	0.26	
		2 years	0.28 (1.10) [328]	0.50 (1.06) [323]	-0.21 (-0.36 to -0.06)	0.00		
		3 years	0.33 (1.06) [360]	0.50 (1.07) [365]	-0.17 (-0.32 to -0.03)	0.02		
By calcium intake[2]	<median	1 year	0.15 (1.01) [314]	0.15 (1.03) [331]	-0.01 (-0.17 to 0.14)	0.86	0.16	0.08
		2 years	0.18 (1.08) [288]	0.21 (1.34) [310]	-0.03 (-0.19 to 0.13)	0.75		
		3 years	0.21 (1.07) [312]	0.20 (1.14) [362]	0.01 (-0.14 to 0.17)	0.87		
	≥median	1 year	0.18 (1.04) [327]	0.22 (1.01) [314]	-0.04 (-0.21 to 0.13)	0.61	0.27	
		2 years	0.13 (1.67) [307]	0.31 (1.07) [279]	-0.15 (-0.32 to 0.03)	0.10		
		3 years	0.15 (1.07) [336]	0.22 (1.23) [311]	-0.09 (-0.26 to 0.08)	0.31		
By baseline 25(OH)D concentration[3]	<75 nmol/L	1 year	0.30 (1.03) [349]	0.28 (0.98) [332]	0.03 (-0.14 to 0.19)	0.76	0.31	0.31
		2 years	0.22 (1.64) [325]	0.31 (1.33) [323]	-0.07 (-0.24 to 0.10)	0.41		
		3 years	0.25 (1.07) [340]	0.34 (1.08) [359]	-0.09 (-0.25 to 0.08)	0.31		
	≥75 nmol/L	1 year	0.02 (0.97) [187]	0.09 (1.02) [201]	-0.06 (-0.25 to 0.13)	0.57	0.58	
		2 years	0.01 (1.05) [171]	0.18 (1.00) [179]	-0.11 (-0.30 to 0.09)	0.28		
		3 years	0.09 (1.00) [196]	0.06 (1.17) [206]	0.02 (-0.17 to 0.21)	0.87		

Abbreviations: 25(OH)D, 25-hydroxyvitamin D. CI, confidence interval. S.d., standard deviation. n, number.

Footnotes. 1, effect estimates and P values from mixed effects linear regression models including a fixed effect for allocation to vitamin D vs. placebo, a random effect for repeated assessments of each individual participant and a random effect of school of attendance. **2,** median calcium intake 466 mg/day. **3,** deseasonalised values.

Confidential: For Review Only

Table 4. End-study pubertal development indices by allocation, sub-study participants: overall and by sub-group

				Vitamin D arm: mean value (s.d.) [n]	Placebo arm: mean value (s.d.) [n]	Adjusted mean difference (95% CI)[1]	P[1]	P for interaction[1]
Males	Pubic hair, mean Tanner score (s.d.) [n]	Overall		3.15 (0.91) [96]	2.81 (0.99) [90]	0.25 (-0.02 to 0.51)	0.07	--
		Calcium intake[2]	<median	3.13 (0.93) [46]	2.73 (1.14) [52]	0.29 (-0.10 to 0.67)	0.14	0.39
			≥median	3.15 (0.90) [48]	2.92 (0.75) [38]	0.16 (-0.19 to 0.52)	0.37	
		Baseline 25(OH)D concentration[3]	<75 nmol/L	3.11 (0.92) [44]	2.88 (0.92) [42]	0.07 (-0.28 to 0.41)	0.70	0.39
			≥75 nmol/L	3.18 (0.94) [34]	2.70 (1.18) [30]	0.43 (-0.07 to 0.93)	0.09	
	External genitalia, mean Tanner score (s.d.) [n]	Overall		3.19 (0.91) [96]	2.90 (0.99) [90]	0.17 (-0.09 to 0.43)	0.19	--
		Calcium intake[2]	<median	3.28 (0.89) [46]	2.81 (1.10) [52]	0.35 (-0.01 to 0.71)	0.06	0.07
			≥median	3.10 (0.93) [48]	3.03 (0.82) [38]	-0.04 (-0.40 to 0.33)	0.84	
		Baseline 25(OH)D concentration[3]	<75 nmol/L	3.27 (0.82) [44]	2.90 (0.91) [42]	0.22 (-0.10 to 0.54)	0.18	0.81
			≥75 nmol/L	3.15 (1.02) [34]	2.87 (1.17) [30]	0.30 (-0.17 to 0.77)	0.21	
Females	Pubic hair, mean Tanner score (s.d.) [n]	Overall		3.10 (0.98) [105]	3.11 (0.98) [99]	-0.09 (-0.35 to 0.17)	0.50	--
		Calcium intake[2]	<median	3.15 (0.98) [47]	3.15 (1.01) [46]	0.01 (-0.38 to 0.40)	0.96	0.60
			≥median	3.11 (0.92) [54]	3.10 (0.97) [50]	-0.23 (-0.56 to 0.10)	0.17	
		Baseline 25(OH)D concentration[3]	<75 nmol/L	3.14 (0.90) [59]	3.13 (0.88) [55]	-0.03 (-0.35 to 0.28)	0.83	0.94
			≥75 nmol/L	2.96 (1.22) [26]	3.04 (1.08) [24]	-0.14 (-0.74 to 0.46)	0.65	
	Breast development, mean Tanner score (s.d.) [n]	Overall		3.15 (0.87) [105]	3.10 (0.91) [99]	-0.03 (-0.26 to 0.20)	0.80	--
		Calcium intake[2]	<median	3.15 (0.91) [47]	3.22 (0.92) [46]	-0.06 (-0.41 to 0.29)	0.74	0.93
			≥median	3.20 (0.81) [54]	3.04 (0.90) [50]	-0.05 (-0.36 to 0.26)	0.76	
		Baseline 25(OH)D concentration[3]	<75 nmol/L	3.27 (0.85) [59]	3.07 (0.86) [55]	0.15 (-0.16 to 0.45)	0.34	0.48
			≥75 nmol/L	3.08 (0.93) [26]	3.08 (0.97) [24]	-0.08 (-0.55 to 0.38)	0.73	
	Proportion menstruating	Overall		67/105 (63.81)	60/99 (60.61)	0.95 (0.51 to 1.77)	0.86	--
		Calcium intake[2]	<median	28/47 (59.57)	26/46 (56.52)	1.33 (0.50 to 3.56)	0.57	0.24
			≥median	38/54 (70.37)	34/50 (68.00)	0.56 (0.21 to 1.52)	0.26	
		Baseline 25(OH)D concentration[3]	<75 nmol/L	43/59 (72.88)	35/55 (63.64)	1.39 (0.59 to 3.28)	0.45	0.59
			≥75 nmol/L	16/26 (61.54)	15/24 (62.50)	0.90 (0.26 to 3.15)	0.87	
	Mean age at menarche, years (s.d.) [n]	Overall		11.94 (0.93) [66]	11.86 (0.66) [58]	-0.03 (-0.31 to 0.25)	0.84	--
		Calcium intake[2]	<median	11.89 (0.83) [28]	11.71 (0.69) [24]	0.12 (-0.26 to 0.49)	0.54	0.32
			≥median	11.97 (1.01) [37]	11.97 (0.63) [34]	-0.11 (-0.51 to 0.29)	0.58	
		Baseline 25(OH)D concentration[3]	<75 nmol/L	12.00 (0.88) [42]	11.88 (0.69) [34]	0.04 (-0.31 to 0.39)	0.82	0.64
			≥75 nmol/L	11.88 (1.15) [16]	11.93 (0.62) [14]	-0.14 (-0.73 to 0.44)	0.63	

Abbreviations: 25(OH)D, 25-hydroxyvitamin D. CI, confidence interval. S.d., standard deviation. n, number.

Footnotes. 1, effect estimates and P values from multi-level mixed models including a random effect for school of attendance. **2,** median calcium intake 466 mg/day. **3,** deseasonalised values.

Confidential: For Review Only

Figure Legend

Figure 1. Participant Flow Diagram

Confidential: For Review Only

References

1. Eccles, J.S., *The development of children ages 6 to 14.* Future Child, 1999. **9**(2): p. 30-44.
2. Dewey, K.G. and K. Begum, *Long-term consequences of stunting in early life.* Matern Child Nutr, 2011. **7 Suppl 3**(Suppl 3): p. 5-18.
3. Vaivada, T., et al., *Stunting in childhood: an overview of global burden, trends, determinants, and drivers of decline.* Am J Clin Nutr, 2020. **112**(Suppl 2): p. 777S-791S.
4. Di Cesare, M., et al., *The epidemiological burden of obesity in childhood: a worldwide epidemic requiring urgent action.* BMC Med, 2019. **17**(1): p. 212.
5. Eckert-Lind, C., et al., *Worldwide Secular Trends in Age at Pubertal Onset Assessed by Breast Development Among Girls: A Systematic Review and Meta-analysis.* JAMA Pediatr, 2020. **174**(4): p. e195881.
6. Bikle, D.D., *Vitamin D: Production, Metabolism and Mechanisms of Action*, in *Endotext*, K.R. Feingold, et al., Editors. 2000: South Dartmouth (MA).
7. Gilbert-Diamond, D., et al., *Vitamin D deficiency and anthropometric indicators of adiposity in school-age children: a prospective study.* Am J Clin Nutr, 2010. **92**(6): p. 1446-51.
8. Foo, L.H., et al., *Relationship between vitamin D status, body composition and physical exercise of adolescent girls in Beijing.* Osteoporos Int, 2009. **20**(3): p. 417-25.
9. Zakharova, I., et al., *Vitamin D Insufficiency in Overweight and Obese Children and Adolescents.* Front Endocrinol (Lausanne), 2019. **10**: p. 103.
10. Plesner, J.L., et al., *Obesity is associated with vitamin D deficiency in Danish children and adolescents.* J Pediatr Endocrinol Metab, 2018. **31**(1): p. 53-61.
11. Theodoratou, E., et al., *Vitamin D and multiple health outcomes: umbrella review of systematic reviews and meta-analyses of observational studies and randomised trials.* BMJ, 2014. **348**: p. g2035.
12. Lee, H.S., et al., *Associations between serum vitamin D levels and precocious puberty in girls.* Ann Pediatr Endocrinol Metab, 2014. **19**(2): p. 91-5.
13. Liu, S., et al., *The association between vitamin D levels and precocious puberty: a meta-analysis.* J Pediatr Endocrinol Metab, 2020. **33**(3): p. 427-429.
14. Gou, Z., et al., *Causal associations between insulin-like growth factor 1 and vitamin D levels: a two-sample bidirectional Mendelian randomization study.* Front Nutr, 2023. **10**: p. 1162442.
15. Nimitphong, H., E. Park, and M.J. Lee, *Vitamin D regulation of adipogenesis and adipose tissue functions.* Nutr Res Pract, 2020. **14**(6): p. 553-567.
16. Ganmaa, D., et al., *Vitamin D supplementation and growth in urban Mongol school children: Results from two randomized clinical trials.* PLoS One, 2017. **12**(5): p. e0175237.
17. Ganmaa, D., et al., *Influence of Vitamin D Supplementation on Growth, Body Composition, and Pubertal Development Among School-aged Children in an Area With a High Prevalence of Vitamin D Deficiency: A Randomized Clinical Trial.* JAMA Pediatr, 2023. **177**(1): p. 32-41.
18. Middelkoop, K., et al., *Vitamin D Supplementation to Prevent Tuberculosis Infection in South African Schoolchildren: Multicentre Phase 3 Double-Blind Randomised Placebo-Controlled Trial (ViDiKids).* Int J Infect Dis, 2023. **134**: p. 63-70.
19. Middelkoop, K., et al., *Influence of vitamin D supplementation on bone mineral content, bone turnover markers and fracture risk in South African schoolchildren:*

Confidential: For Review Only

multicentre double-blind randomised placebo-controlled trial (ViDiKids). J Bone Miner Res https://doi.org/10.1093/jbmr/zjae007, 2024.
20. Graham, B.L., et al., *Standardization of Spirometry 2019 Update. An Official American Thoracic Society and European Respiratory Society Technical Statement.* Am J Respir Crit Care Med, 2019. **200**(8): p. e70-e88.
21. Rajakumar, K., et al., *Estimations of dietary vitamin D requirements in black and white children.* Pediatr Res, 2016. **80**(1): p. 14-20.
22. Iglay, K., et al., *Systematic Literature Review and Meta-analysis of Medication Adherence With Once-weekly Versus Once-daily Therapy.* Clin Ther, 2015. **37**(8): p. 1813-21 e1.
23. El-Hajj Fuleihan, G., et al., *Effect of vitamin D replacement on musculoskeletal parameters in school children: a randomized controlled trial.* J Clin Endocrinol Metab, 2006. **91**(2): p. 405-12.
24. Chavarro, J.E., et al., *Validity of Self-Assessed Sexual Maturation Against Physician Assessments and Hormone Levels.* J Pediatr, 2017. **186**: p. 172-178 e3.
25. Middelkoop, K., et al., *Prevalence and Determinants of Vitamin D Deficiency in 1825 Cape Town Primary Schoolchildren: A Cross-Sectional Study.* Nutrients, 2022. **14**(6): p. 1263.
26. Ganmaa, D., et al., *Prevalence and Determinants of QuantiFERON-Diagnosed Tuberculosis Infection in 9810 Mongolian Schoolchildren.* Clin Infect Dis, 2019. **69**(5): p. 813-819.
27. Norris, S.A. and L.M. Richter, *Usefulness and Reliability of Tanner Pubertal Self-Rating to Urban Black Adolescents in South Africa.* Journal of Research on Adolescence, 2005. **15**: p. 609-624.

Confidential: For Review Only

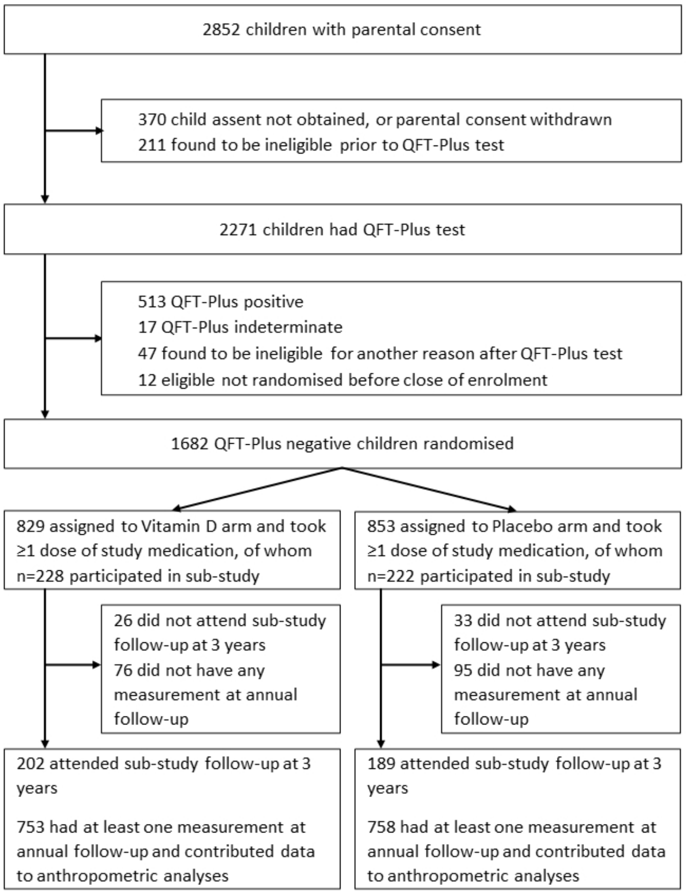

Figure 1. Participant Flow Diagram

229x300mm (96 x 96 DPI)

Influence of vitamin D supplementation on growth, body composition, pubertal development and spirometry in South African schoolchildren: a randomised controlled trial (ViDiKids)

Supplemental Material

Confidential: For Review Only

Contents

Supplementary Methods 2
Enrolment 2
Randomisation and blinding 2
Intervention 4
Laboratory Assessments 5
Statistical Analyses 5
Table S1. Mean waist circumference-for-age z-scores at annual follow-up by allocation, main trial participants: overall and by sub-group 7
Table S2. Mean waist-to-height ratio z-scores at annual follow-up by allocation, main trial participants: overall and by sub-group 8
Table S3. Fat mass and fat-free soft tissue mass at 3-year follow-up by allocation, sub-study participants: overall and by sub-group 9
Table S4. Spirometric outcomes at 3-year follow-up by allocation, sub-study participants: overall and by sub-group 10

Supplementary Methods

Enrolment

Parents or legal guardians were invited to provide written informed consent for their child to participate in the main trial during a home visit, unless their child was eligible for the sub-study, in which case they were invited to provide written informed consent for their child to participate in both the main trial and the sub-study until a total of 450 sub-study participants were randomised. If parents / legal guardians consented, they were asked to provide details of their child's dietary intake of foods containing vitamin D and calcium in the previous month, which were captured on an electronic case report form as previously reported.[19] Their children were then invited to provide written assent to participate in the main trial +/- the sub-study (if eligible) at a school-based visit. If they agreed, a clinically trained member of the study team screened them for symptoms and signs of rickets. For all participants, a blood sample was taken for a QFT-Plus assay and separation and storage of serum for determination of 25(OH)D concentrations as described below. Participants were reviewed when baseline QFT-Plus results were available. Those with a positive QFT-Plus result were excluded from the trial and screened for active TB. Those with an indeterminate QFT-Plus result were excluded from the trial without screening for active TB. Those with a negative QFT-Plus result were deemed eligible to participate and underwent measurement of weight (using a digital floor scale, Charder Medical, Taichung City, Taiwan), height (using a portable HM200P stadiometer, Charder Medical) and waist circumference (using a measuring tape). Sub-study participants also underwent spirometry according to ERS/ATS standards[20] using a portable spirometer (Carefusion, San Diego, CA) and measurement of body composition by dual energy x-ray absorptiometry (DXA) as described below.

Randomisation and blinding

Eligible participants were individually randomised to receive a weekly capsule containing vitamin D_3 or placebo for three years, with a one-to-one allocation ratio. Randomisation was stratified by school of attendance (a potential predictor of risk of QFT conversion) as follows. Prior to the start of recruitment, Mrs Claire Chan

Confidential: For Review Only

(Independent statistician, Pragmatic Clinical Trials Unit, Queen Mary University of London) prepared two school randomisation lists, each comprising 20 pairs of 2-letter randomisation codes, with each pair allocated to a single school identifier (e.g. School 1 was allocated codes 'AC' and 'MO', School 2 was allocated codes 'BD' and 'NP'). One 2-letter randomisation code within each pair was then randomly assigned to the vitamin D arm of the trial, and the other was assigned to the placebo arm, using a computer-generated random sequence (e.g. 'AC' and 'BD' were assigned to 'vitamin D', 'MO' and 'NP' were assigned to placebo). Mrs Chan also prepared 40 separate participant randomisation lists (one for each potential participating school). Each of these participant randomisation lists comprised 999 5-digit numbers, each consisting of a 2-digit school identifier from 01 to 25 that was constant for, and unique to, each list, followed by a 3-digit participant identifier from 001 to 999 (e.g. 01-001, 01-002, 02-001, 02-002). These 5-digit numbers were then randomly assigned to one or other of the 2-letter randomisation codes allocated to that school in blocks of ten, using a computer-generated random sequence (e.g. for School 1, sequence was 01-001-MO, 01-002-AC, etc.; for School 2, sequence was 02-001-BD, 02-002-BD, etc.).

Active and placebo capsules were shipped from Tishcon Corporation (Westbury, NY, USA) to Lekoko Pharmaceutical Management Consultancy (Johannesburg, South Africa) in boxes that were labelled 'vitamin D' or 'placebo' according to their contents. On arrival, these capsules were packed into bottles, each containing either 150 vitamin D capsules or 150 placebo capsules. The school randomisation list was then used to label these bottles with 2-letter randomisation codes according to their contents (i.e. bottles containing vitamin D capsules were labelled with 'AC', 'BD' or another 2-letter code assigned to the vitamin D arm of the trial, while bottles containing placebo capsules were labelled 'MO', 'NP' or another 2-letter code assigned to the placebo arm of the trial) until sufficient capsules for 23 schools had been bottled and labelled. Bottling and labelling was performed under the supervision of Mr Bobby Hamman (Lekoko PMC), with independent monitoring performed by staff from OnQ Contract Research Organisation; none of these individuals was involved with data collection. Children screened at each school were assigned consecutive 5-digit numbers at enrolment by study field workers, and if they were subsequently found to be eligible for randomisation (i.e. if their baseline QFT-Plus result was negative) then the participant randomisation list for their school of attendance was used to determine

Confidential: For Review Only

their allocation, i.e. they received study medication from bottles labelled with the 2-letter code linked to their 5-digit ID in the participant randomization list for the duration of the trial. For example, participant 01-001 would receive study medication labelled ‘MO’ (i.e. placebo), 01-002 would receive medication labelled ‘AC’ (i.e. vitamin D), 02-001 would receive medication labelled ‘BD’ (vitamin D), and 02-002 would receive medication labelled ‘BD’ (vitamin D) throughout the trial. Copies of the school randomisation list were held by Mrs Chan and by members of the DSMB. Neither participants nor trial staff had access to it, and treatment allocation was concealed from participants, care providers and all trial staff (including senior investigators and those assessing outcomes) so that the double-blind was maintained. The school randomisation lists were made available to Dr Neil Walker (trial statistician) following completion of the trial, who used them to un-blind allocation and to analyse the trial: he was not therefore masked to group assignment during statistical analysis.

Intervention

Study medication comprised a 3-year course of weekly soft gel capsules manufactured by the Tishcon Corporation (Westbury, NY, USA), containing either 0.25 mg (10,000 international units) cholecalciferol (vitamin D_3) in olive oil (intervention arm) or olive oil without any vitamin D_3 content (placebo arm). Active and placebo capsules had identical appearance and taste. Capsules were taken under direct observation of study staff during school term time. Further details of administration of study medication are provided in Supplementary Material. During summer holidays (8 weeks), packs containing 8 doses of study medication were provided for administration by parents, together with a participant diary. Following shorter school holidays (≤4 weeks), and/or if participants missed one or more doses of study medication during term time, up to 4 ‘catch-up’ doses were administered at the first weekly visit attended following the missed dose(s). During the initial national lockdown for COVID-19 in South Africa (27th March to 1st May 2020), participants did not receive any study medication. During subsequent school closures due to COVID-19, two rounds of 8-week holiday packs were provided to participants, which were sufficient to cover their requirements until schools re-opened.

Confidential: For Review Only

Laboratory Assessments

Biochemical analyses were performed at the Bioanalytical Facility, University of East Anglia (Norwich, UK) according to manufacturers' instructions and under Good Clinical and Laboratory Practice conditions. Serum concentrations of 25(OH)D_3 were measured using liquid chromatography tandem mass spectrometry (LC-MS/MS) as previously described.[22] 25(OH)D_3 was calibrated using standard reference material SRM972a from the National Institute of Science and Technology (NIST), and the assay showed linearity between 0 and 200 nmol/L. The inter/intra-assay coefficient of variation (CV) across the assay range was ≤9%, and the lower limit of quantification was 0.1 nmol/L. The assay showed <6% accuracy bias against NIST reference method on the vitamin D external quality assessment (DEQAS) scheme (http://www.deqas.org/; accessed on 30th November 2022). QFT-Plus assays were performed by the Bio Analytical Research Corporation South Africa (Johannesburg, South Africa) according to the manufacturer's instructions.

Statistical Analyses

Statistical analyses were performed using Stata software (Version 17.0; StataCorp, College Station, Texas, United States) according to intention to treat. Anthropometric measurements were used to compute age- and sex-adjusted z-scores for height, BMI, waist circumference and waist-to-height ratio, using the WHO 2007 Lambda, Mu and Sigma method.[23] Effects of treatment on the above Z-transformed outcomes were estimated by fitting allocation to vitamin D vs. placebo as the sole fixed effect in a mixed effects linear regression model with a random effect for repeated assessments of each individual participant (at baseline and years 1, 2 and 3 of follow-up) and a random effect of school of attendance, with results reported as treatment differences with 95% confidence intervals. Effects of treatment on DXA and spirometric outcomes measured at 3-year follow-up were analysed in each case using a multi-level mixed model with an adjustment for analogous baseline score and a random effect for school. Tanner metrics on pubertal development were analysed in a similar fashion, but

Confidential: For Review Only

restricted to the applicable sex and without baseline adjustment. Binary outcomes measured at 3-year follow-up only were analysed using a mixed-effects logistic regression model, with treatment allocation as the sole fixed effect and school attended as a random intercept. Pre-specified sub-group analyses were conducted to determine whether the effect of vitamin D supplementation was modified by sex (male vs. female), baseline deseasonalised 25(OH)D_3 concentration (<75 vs. ≥75 nmol/L, calculated using a sinusoidal model as previously described)[24] and calcium intake (< vs. ≥ median value of 466 mg/day, calculated from a dietary intake questionnaire as previously described).[19] These were performed by repeating efficacy analyses with the inclusion of an interaction term between allocation (to vitamin D vs. placebo) and each posited effect-modifier with presentation of the P-value associated with this interaction term. Effect of sex as a subgroup was not considered in relation to analyses of Tanner outcomes, as these are sex-specific. Given the number of potential effect modifiers and secondary outcome measures these analyses are considered exploratory. Interim safety assessments, where Independent Data Monitoring Committee (IDMC) members reviewed accumulating serious adverse event data, were performed at 6-monthly intervals. At each review, the IDMC recommended continuation of the trial. No interim efficacy analysis was performed.

Confidential: For Review Only

Table S1. Mean waist circumference-for-age z-scores at annual follow-up by allocation, main trial participants: overall and by sub-group

			Vitamin D: mean value (s.d.) [n]	Placebo: mean value (s.d.) [n]	Adjusted mean difference (95% CI)[1]	P for timepoint[1]	P for trend[1]	P for interaction (treatment*subgroup)[1]
Overall		1 year	-0.03 (0.89) [664]	-0.00 (0.87) [660]	-0.03 (-0.12 to 0.06)	0.47	0.01	
		2 years	0.19 (0.87) [612]	0.24 (0.87) [608]	-0.06 (-0.15 to 0.04)	0.24		
		3 years	0.15 (0.83) [667]	0.21 (0.88) [686]	-0.07 (-0.16 to 0.02)	0.12		
By sex	Boys	1 year	-0.14 (0.84) [304]	-0.20 (0.88) [305]	0.06 (-0.07 to 0.19)	0.39	0.07	0.93
		2 years	0.11 (0.84) [286]	0.06 (0.88) [284]	0.05 (-0.08 to 0.18)	0.47		
		3 years	0.03 (0.83) [309]	0.03 (0.85) [325]	-0.02 (-0.15 to 0.11)	0.78		
	Girls	1 year	0.06 (0.92) [360]	0.17 (0.82) [355]	-0.12 (-0.24 to 0.00)	0.05	0.04	
		2 years	0.26 (0.88) [326]	0.41 (0.84) [324]	-0.15 (-0.28 to -0.03)	0.01		
		3 years	0.25 (0.82) [358]	0.37 (0.86) [361]	-0.13 (-0.25 to -0.01)	0.04		
By calcium intake[2]	<median	1 year	-0.04 (0.89) [314]	-0.05 (0.86) [331]	0.00 (-0.12 to 0.13)	0.96	0.02	0.64
		2 years	0.19 (0.86) [289]	0.19 (0.89) [312]	0.00 (-0.12 to 0.13)	0.94		
		3 years	0.16 (0.81) [310]	0.21 (0.85) [358]	-0.03 (-0.16 to 0.09)	0.62		
	≥median	1 year	-0.01 (0.89) [327]	0.06 (0.89) [313]	-0.07 (-0.20 to 0.06)	0.28	0.10	
		2 years	0.20 (0.87) [305]	0.30 (0.86) [279]	-0.10 (-0.23 to 0.03)	0.14		
		3 years	0.14 (0.87) [337]	0.22 (0.91) [310]	-0.11 (-0.24 to 0.02)	0.10		
By baseline 25(OH)D concentration[3]	<75 nmol/L	1 year	0.09 (0.90) [349]	0.08 (0.87) [332]	0.02 (-0.10 to 0.15)	0.72	0.08	0.68
		2 years	0.26 (0.89) [324]	0.31 (0.88) [324]	-0.02 (-0.15 to 0.11)	0.75		
		3 years	0.21 (0.85) [338]	0.29 (0.85) [356]	-0.06 (-0.18 to 0.06)	0.34		
	≥75 nmol/L	1 year	-0.19 (0.84) [187]	-0.06 (0.85) [200]	-0.14 (-0.30 to 0.02)	0.08	0.07	
		2 years	0.05 (0.81) [171]	0.20 (0.82) [179]	-0.15 (-0.31 to 0.01)	0.07		
		3 years	0.03 (0.76) [196]	0.14 (0.87) [205]	-0.11 (-0.26 to 0.05)	0.19		

Abbreviations: 25(OH)D, 25-hydroxyvitamin D. CI, confidence interval. S.d., standard deviation. n, number.

Footnotes. 1, effect estimates and P values from mixed effects linear regression models including a fixed effect for allocation to vitamin D vs. placebo, a random effect for repeated assessments of each individual participant and a random effect of school of attendance. **2,** median calcium intake 466 mg/day. **3,** deseasonalised values.

Confidential: For Review Only

Table S2. Mean waist-to-height ratio z-scores at annual follow-up by allocation, main trial participants: overall and by sub-group

			**Vitamin D: mean value (s.d.) [n]**	**Placebo: mean value (s.d.) [n]**	**Adjusted mean difference (95% CI)[1]**	**P for timepoint[1]**	**P for trend[1]**	**P for interaction (treatment*subgroup)[1]**
Overall		1 year	-0.01 (0.82) [664]	-0.01 (0.81) [659]	-0.02 (-0.10 to 0.07)	0.67		
		2 years	0.15 (0.83) [610]	0.19 (0.83) [606]	-0.04 (-0.13 to 0.04)	0.32	0.01	
		3 years	0.11 (0.80) [667]	0.15 (0.83) [686]	-0.05 (-0.14 to 0.03)	0.22		
By sex	Boys	1 year	-0.04 (0.76) [304]	-0.12 (0.80) [304]	0.06 (-0.07 to 0.18)	0.36		
		2 years	0.17 (0.76) [284]	0.12 (0.78) [283]	0.03 (-0.09 to 0.16)	0.60	0.08	
		3 years	0.09 (0.78) [309]	0.08 (0.78) [325]	-0.01 (-0.13 to 0.11)	0.85		
	Girls	1 year	0.02 (0.87) [360]	0.09 (0.80) [355]	-0.09 (-0.21 to 0.03)	0.14		
		2 years	0.15 (0.88) [326]	0.26 (0.86) [323]	-0.12 (-0.24 to 0.00)	0.06	0.08	0.83
		3 years	0.13 (0.82) [358]	0.21 (0.87) [361]	-0.10 (-0.21 to 0.02)	0.11		
By calcium intake[2]	<median	1 year	-0.05 (0.81) [314]	-0.09 (0.82) [331]	0.02 (-0.10 to 0.14)	0.71		
		2 years	0.17 (0.79) [288]	0.13 (0.84) [310]	0.02 (-0.11 to 0.14)	0.77	0.03	
		3 years	0.13 (0.78) [310]	0.15 (0.80) [358]	-0.02 (-0.14 to 0.10)	0.74		
	≥median	1 year	0.01 (0.85) [327]	0.08 (0.80) [312]	-0.08 (-0.20 to 0.05)	0.22		
		2 years	0.15 (0.85) [304]	0.25 (0.82) [279]	-0.10 (-0.23 to 0.03)	0.12	0.15	0.64
		3 years	0.09 (0.83) [337]	0.15 (0.88) [310]	-0.09 (-0.21 to 0.04)	0.16		
By baseline 25(OH)D concentration[3]	<75 nmol/L	1 year	0.11 (0.82) [349]	0.04 (0.80) [331]	0.08 (-0.04 to 0.19)	0.22		
		2 years	0.23 (0.84) [323]	0.21 (0.83) [323]	0.03 (-0.09 to 0.15)	0.66	0.07	
		3 years	0.17 (0.82) [338]	0.19 (0.80) [356]	-0.02 (-0.14 to 0.10)	0.78		
	≥75 nmol/L	1 year	-0.17 (0.80) [187]	-0.04 (0.83) [200]	-0.15 (-0.44 to 0.15)	0.33	0.15	
		2 years	0.02 (0.76) [170]	0.16 (0.77) [179]	-0.15 (-0.46 to 0.16)	0.34		0.88
		3 years	0.01 (0.75) [196]	0.09 (0.81) [205]	-0.10 (-0.39 to 0.19)	0.51		

Abbreviations: 25(OH)D, 25-hydroxyvitamin D. CI, confidence interval. S.d., standard deviation. n, number.

Footnotes. 1, effect estimates and P values from mixed effects linear regression models including a fixed effect for allocation to vitamin D vs. placebo, a random effect for repeated assessments of each individual participant and a random effect of school of attendance. **2,** median calcium intake 466 mg/day. **3,** deseasonalised values.

Confidential: For Review Only

Table S3. Fat mass and fat-free soft tissue mass at 3-year follow-up by allocation, sub-study participants: overall and by sub-group

		Vitamin D: mean value (s.d.) [n]	Placebo: mean value (s.d.) [n]	Adjusted mean difference (95% CI)[1]	P[1]	P for interaction[1]
**Fat mass, kg**						
Overall		6.04 (3.86) [202]	6.28 (3.99) [189]	-0.15 (-0.51 to 0.20)	0.40	
By sex	Boys	4.80 (3.11) [97]	4.78 (3.05) [89]	0.18 (-0.30 to 0.66)	0.45	0.14
	Girls	7.19 (4.13) [105]	7.61 (4.27) [100]	-0.45 (-0.96 to 0.05)	0.08	
By calcium intake[2]	<median	5.73 (3.32) [94]	5.74 (3.55) [97]	0.04 (-0.42 to 0.49)	0.88	0.34
	≥median	6.41 (4.35) [102]	6.88 (4.41) [89]	-0.34 (-0.90 to 0.23)	0.24	
By baseline 25(OH)D concentration[3]	<75 nmol/L	6.74 (4.28) [103]	6.64 (4.23) [96]	-0.32 (-0.85 to 0.21)	0.24	0.54
	≥75 nmol/L	5.39 (3.47) [60]	5.85 (3.71) [54]	-0.04 (-0.62 to 0.55)	0.91	
By baseline fat mass	<median	3.72 (1.08) [103]	3.65 (1.20) [90]	0.02 (-0.24 to 0.27)	0.88	0.42
	≥median	8.46 (4.20) [99]	8.67 (4.15) [99]	-0.27 (-0.91 to 0.37)	0.41	
**Fat-free soft tissue mass, kg**						
Overall		13.22 (2.87) [202]	13.07 (2.70) [189]	-0.13 (-0.42 to 0.15)	0.36	
By sex	Boys	14.00 (2.87) [97]	13.54 (3.03) [89]	-0.01 (-0.43 to 0.41)	0.96	0.08
	Girls	12.50 (2.69) [105]	12.64 (2.29) [100]	-0.28 (-0.63 to 0.06)	0.10	
By calcium intake[2]	<median	13.31 (2.68) [94]	13.08 (2.67) [97]	-0.09 (-0.48 to 0.30)	0.67	0.68
	≥median	13.23 (3.07) [102]	13.16 (2.69) [89]	-0.16 (-0.59 to 0.27)	0.48	
By baseline 25(OH)D concentration[3]	<75 nmol/L	13.33 (3.06) [103]	13.33 (2.79) [96]	-0.21 (-0.62 to 0.21)	0.33	0.91
	≥75 nmol/L	13.30 (3.03) [60]	13.01 (2.72) [54]	-0.17 (-0.68 to 0.34)	0.51	
By baseline fat-free soft tissue mass	<median	11.06 (1.54) [89]	11.40 (1.53) [103]	-0.13 (-0.43 to 0.16)	0.38	0.95
	≥median	14.92 (2.52) [113]	15.06 (2.43) [86]	-0.14 (-0.60 to 0.33)	0.57	

Abbreviations: 25(OH)D, 25-hydroxyvitamin D. CI, confidence interval. S.d., standard deviation. n, number.
Footnotes. 1, effect estimates and P values from multi-level mixed models with adjustment for baseline values and a random effect for school of attendance. **2,** median calcium intake 466 mg/day. **3,** deseasonalised values.

Confidential: For Review Only

Table S4. Spirometric outcomes at 3-year follow-up by allocation, sub-study participants: overall and by sub-group

		Vitamin D: mean value (s.d.) [n]	Placebo: mean value (s.d.) [n]	Adjusted mean difference (95% CI)[1]	P[1]	P for interaction[1]
**% predicted FEV1**						
Overall		81.46 (13.37) [200]	82.48 (14.54) [187]	-1.68 (-4.23 to 0.88)	0.20	
By sex	Boys	80.33 (11.53) [95]	83.15 (12.94) [89]	-2.95 (-6.19 to 0.29)	0.07	0.16
	Girls	82.48 (14.81) [105]	81.87 (15.90) [98]	-0.50 (-4.30 to 3.31)	0.80	
By calcium intake[2]	<median	82.97 (12.53) [92]	83.31 (15.19) [97]	-1.40 (-4.98 to 2.18)	0.44	0.95
	≥median	80.58 (13.85) [102]	81.79 (13.90) [87]	-1.44 (-5.21 to 2.33)	0.45	
By baseline 25(OH)D concentration[3]	<75 nmol/L	82.26 (11.99) [103]	82.98 (14.21) [95]	-0.34 (-3.72 to 3.05)	0.84	0.10
	≥75 nmol/L	80.10 (12.14) [59]	84.34 (14.23) [54]	-4.80 (-9.12 to -0.49)	0.03	
**% predicted FVC**						
Overall		86.74 (13.53) [200]	87.10 (13.51) [187]	0.23 (-2.30 to 2.76)	0.86	
By sex	Boys	87.77 (12.16) [95]	90.36 (11.64) [89]	-2.17 (-5.37 to 1.03)	0.18	0.049
	Girls	85.81 (14.65) [105]	84.13 (14.43) [98]	2.11 (-1.73 to 5.95)	0.28	
By calcium intake[2]	<median	87.51 (12.47) [92]	87.15 (13.86) [97]	0.14 (-3.40 to 3.68)	0.94	0.97
	≥median	86.06 (14.05) [102]	87.19 (13.33) [87]	0.62 (-3.08 to 4.33)	0.74	
By baseline 25(OH)D concentration[3]	<75 nmol/L	86.67 (12.90) [103]	87.19 (13.84) [95]	-0.22 (-3.73 to 3.30)	0.90	0.92
	≥75 nmol/L	87.08 (12.79) [59]	86.85 (11.53) [54]	0.61 (-3.42 to 4.65)	0.77	
**% predicted FEV1/FVC**						
Overall		96.98 (14.02) [200]	96.99 (10.60) [187]	-0.84 (-3.30 to 1.62)	0.50	
By sex	Boys	97.31 (15.28) [95]	96.86 (10.03) [89]	0.02 (-3.64 to 3.69)	0.99	0.67
	Girls	96.69 (12.83) [105]	97.12 (11.14) [98]	-1.32 (-4.58 to 1.94)	0.43	
By calcium intake[2]	<median	97.64 (11.71) [92]	97.96 (9.54) [97]	-0.47 (-3.46 to 2.52)	0.76	0.95
	≥median	96.80 (15.07) [102]	96.12 (11.70) [87]	-1.42 (-5.26 to 2.43)	0.47	
By baseline 25(OH)D concentration[3]	<75 nmol/L	97.39 (10.36) [103]	97.39 (10.73) [95]	-0.38 (-3.26 to 2.50)	0.80	0.16
	≥75 nmol/L	95.63 (13.96) [59]	99.44 (9.48) [54]	-4.47 (-8.82 to -0.12)	0.04	

Abbreviations: 25(OH)D, 25-hydroxyvitamin D. FEV1, forced expiratory volume in 1 second. FVC, forced vital capacity. CI, confidence interval. S.d., standard deviation. n, number.

Footnotes. 1, effect estimates and P values from multi-level mixed models with adjustment for baseline values and a random effect for school of attendance. **2,** median calcium intake 466 mg/day. **3,** deseasonalised values.

Confidential: For Review Only
